# Kelp forest community structure and demography in Kongsfjorden (Svalbard) across 25 years of Arctic warming

**DOI:** 10.1002/ece3.11606

**Published:** 2024-06-25

**Authors:** Luisa Düsedau, Stein Fredriksen, Markus Brand, Philipp Fischer, Ulf Karsten, Kai Bischof, Amanda Savoie, Inka Bartsch

**Affiliations:** ^1^ Alfred Wegener Institute Helmholtz Centre for Polar and Marine Research Bremerhaven Germany; ^2^ Department of Marine Botany University of Bremen & MARUM Bremen Germany; ^3^ Department of Biosciences University of Oslo Oslo Norway; ^4^ Department of Applied Ecology and Phycology Institute of Biological Sciences, University of Rostock Rostock Germany; ^5^ Centre for Arctic Knowledge and Exploration, Canadian Museum of Nature Ottawa Ontario Canada

**Keywords:** age structure, biomass, coastal darkening, depth zonation, macroalgae, time series

## Abstract

The Arctic archipelago of Svalbard is a hotspot of global warming and many fjords experience a continuous increase in seawater temperature and glacial melt while sea‐ice cover declines. In 1996/1998, 2012–2014, and 2021 macroalgal biomass and species diversity were quantified at the study site Hansneset, Kongsfjorden (W‐Spitsbergen) in order to identify potential changes over time. In 2021, we repeated the earlier studies by stratified random sampling (1 × 1 m^2^, *n* = 3) along a sublittoral depth transect (0, 2.5, 5, 10, and 15 m) and investigated the lower depth limits of dominant brown algae between 3 and 19 m. The maximum fresh weight (FW) of all seaweeds was 11.5 kg m^−2^ at 2.5 m and to 99.9% constituted of kelp. Although biomass distribution along the depth transect in 2021 was not significantly different compared to 2012/2013, the digitate kelp community (*Laminaria digitata*/*Hedophyllum nigripes*) had transformed into an *Alaria esculenta*‐dominated kelp forest. Consequently, a pronounced shift in kelp forest structure occurred over time as we demonstrate that biomass allocation to thallus parts is kelp species‐specific. Over the past decade, kelp demography changed and in 2021 a balanced age structure of kelps (juveniles plus many older kelp individuals) was only apparent at 2.5 m. In addition, the abundances and lower depth limits of all dominant brown algae declined noticeably over the last 25 years while the red algal flora abundance remained unchanged at depth. We propose that the major factor driving the observed changes in the macroalgal community are alterations in underwater light climate, as in situ data showed increasing turbidity and decreasing irradiance since 2012 and 2017, respectively. As a consequence, the interplay between kelp forest retreat to lower depth levels caused by coastal darkening and potential macroalgal biomass gain with increasing temperatures will possibly intensify in the future with unforeseen consequences for melting Arctic coasts and fjord ecosystem services.

## Graphical abstract

The Arctic archipelago of Svalbard is a hotspot of global warming and many fjords experience a continuous increase in seawater temperature and glacial melt while sea‐ice cover declines. To document variations in Arctic kelp forest structure and demography the macroalgal community at our sampling site in Kongsfjorden (W‐Spitzbergen) was sampled in a standardized manner at three time points spanning over the past 25 years. Our results imply that the interplay between kelp forest retreat to lower depth levels caused by coastal darkening and potential macroalgal biomass gain with increasing temperatures will possibly intensify in the future with unforeseen consequences for melting Arctic coasts and fjord ecosystem services.

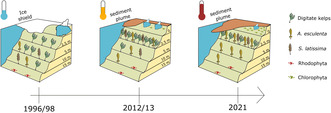

## 1 INTRODUCTION

Submarine forests are present in coastal areas worldwide and predominantly constituted of canopy‐forming algae species (Pessarrodona et al., 2022). In temperate to polar regions many of these forest communities are shaped by large kelps of the brown algal order Laminariales (Dayton, 1985; Lüning, 1990). Kelps serve as important foundation species and major primary producers on rocky shores (Filbee‐Dexter et al., 2019). They contribute strongly to the net primary production and the coastal carbon cycle as carbon is fixed in their biomass through photosynthesis (Krause‐Jensen & Duarte, 2016; Pessarrodona et al., 2022; Smale, Pessarrodona et al., 2022). Overall, kelp forests are particularly valuable ecosystems that host a high biodiversity of marine life and complex food webs (Elliott Smith & Fox, 2022; Smale et al., 2013; Steneck et al., 2002; Teagle et al., 2017). The northward extension of kelp forests into the Arctic is physically limited by sea ice and low light conditions whereas their distribution towards the equator is restricted by nutrient availability and warm temperatures (Steneck & Johnson, 2013).

Caused by ubiquitous global climate change, the Arctic Ocean has warmed four times faster than the global average since 1979 (Rantanen et al., 2022). Consequently, Arctic sea ice coverage has decreased continuously while the open water period expanded (Barnhart et al., 2016). Kelp production will either expand or decrease depending on local environmental settings and species distributions will change in future Arctic habitats (Filbee‐Dexter et al., 2019). Associated changes towards increased underwater light availability with sea ice retreat and reduced physical disturbance by ice scraping are predicted to open up new habitats enabling kelp forest expansion (Assis et al., 2022; Krause‐Jensen & Duarte, 2014). However, the loss of summer ice cover also accelerates wind‐driven coastal erosion in shallow areas, which can increase sediment resuspension and reduce benthic primary production (Bonsell & Dunton, 2018). At the same time, glaciers around the globe are melting rapidly (Hugonnet et al., 2021) and increasing meltwater runoff creates strong turbidity and salinity gradients amplifying the potential negative impacts on Arctic coastal productivity (Jerosch et al., 2019; Sejr et al., 2022).

Kongsfjorden (western Spitsbergen, Svalbard archipelago) is an example of a well‐monitored Arctic fjord that experiences strong impacts of climate change and serves as a marine high‐latitude model ecosystem (Bischof, Convey et al., 2019). The fjord is lined by several glaciers at different stages of glacial retreat with four main calving tidewater glaciers and several land‐terminating glaciers including Brøggerbreen at the Bayelva River (Pavlov et al., 2019; Svendsen et al., 2002). The hydrography of Kongsfjorden is influenced by cold Arctic water from the East Spitsbergen Coastal Current flowing around the Svalbard shelf and warm saline Atlantic water from the West Spitsbergen Current (Svendsen et al., 2002). To the west Kongsfjorden is open to the Arctic Ocean where the water masses entering through the southern part are mixed with fresh water from glacial meltwater and river runoff before flowing out along the northern coast (Kruss et al., 2017).

Reflecting the overall regime shift in the Arctic Ocean (Sumata et al., 2023), warm water masses started to enter Kongsfjorden all year round in 2006 and prevented cold Arctic winters with persistent thick sea ice coverage (Tverberg et al., 2019). Long‐term sea ice monitoring documented this turning point as sea ice thickness and ice extent into the central part of the fjord decreased gradually while the number of ice‐free days increased (Pavlov et al., 2019). Continuous oceanographic measurements suggest that Kongsfjorden has already transitioned to an Atlantic‐type fjord as depth averaged temperatures in the inner fjord in summer have increased by 0.26°C/year since 2010 (De Rovere et al., 2022). When freshwater from glacial melt, snow, and precipitation enters the fjord in summer, it carries suspended terrestrial particles that form large sediment plumes which alter the spectral composition of the underwater light regime available for macroalgal photosynthesis (Niedzwiedz & Bischof, 2023; Pavlov et al., 2019).

The shallow rocky subtidal of the Kongsfjorden coastline down to 15 m depth is densely covered with macroalgal meadows but even at ~70 m depth deep‐water red algae and crustose coralline algae occur (Kruss et al., 2017; Schimani et al., 2022). Along the fjord axis kelp forests experience a gradual alteration in their abiotic environment with effects on the species as well as the community level (Bischof, Buschbaum et al., 2019; Hop et al., 2016; Schimani et al., 2022). In 1996/1998 Hop et al. (2012) investigated for the first time the biodiversity and biomass distribution of macroalgae along a depth transect at the rocky shore of Hansneset. They reported a rich kelp forest that was dominated by digitate kelps (*Laminaria digitata*/*Hedophyllum nigripes*), *Alaria esculenta* and *Saccharina latissima*, and overall documented 62 macroalgal species. In 2012–2014, the hard bottom community was examined a second time and standards for future monitoring were established by Bartsch et al. (2016) and Paar et al. (2016). In the upper subtidal zone (2.5 m) kelp biomass in 2012/2013 was higher compared to 1996/1998 and the study revealed that not only the overall biomass maximum but the entire kelp forest had shallowed (Bartsch et al., 2016). The documented changes in the kelp forest community structure were discussed as being likely a consequence of altering abiotic conditions caused by climate change (Bartsch et al., 2016).

Multi‐stressor experiments indicate that different species and life stages of kelps may benefit from the rapidly changing environmental conditions while others will face fundamental challenges (Diehl & Bischof, 2021; Franke et al., 2021; Niedzwiedz & Bischof, 2023; Roleda, 2016; Zacher et al., 2016). As kelp species have structural differences, shifts in species composition as well as variations in productivity due to Arctic warming ultimately affect associated macrofaunal assemblages (Filbee‐Dexter et al., 2019; Teagle et al., 2017; Włodarska‐Kowalczuk et al., 2009).

**FIGURE 1:**
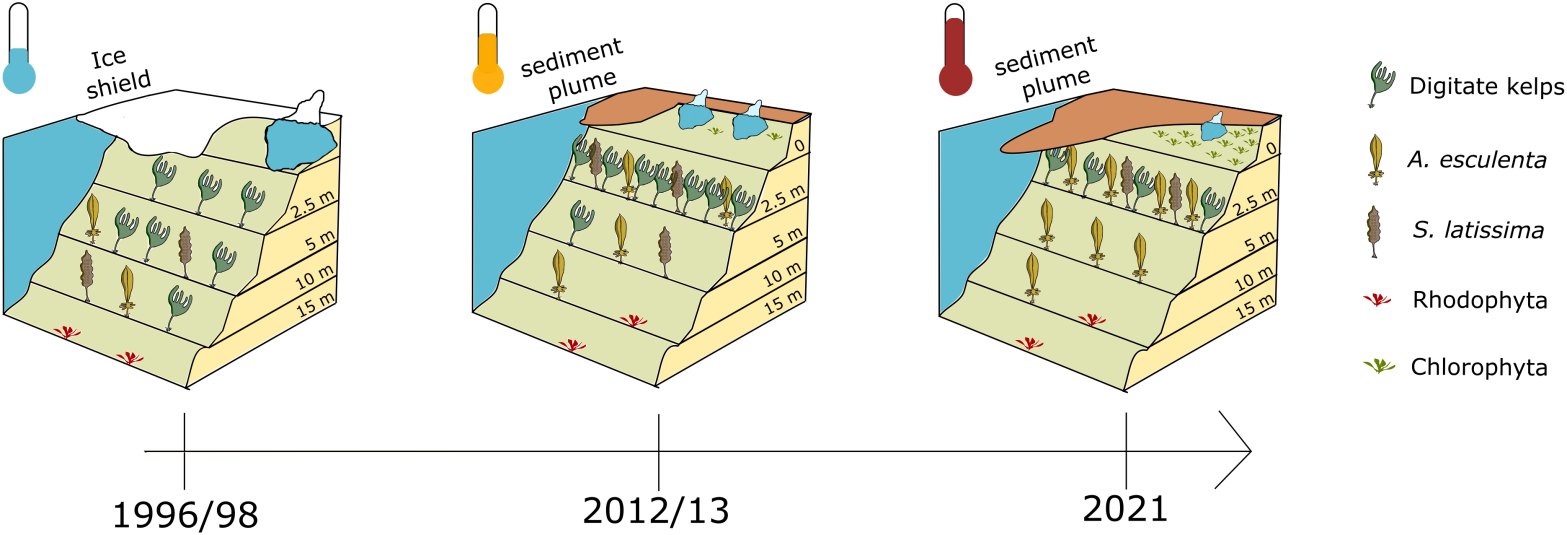
Shift in kelp forest biomass and depth distribution over 25 years of Arctic warming at our study site Hansneset in Kongsfjorden, Svalbard. The habitat for kelp communities along Arctic fjord systems declines as the increase in glacial melt intensifies the phenomenon of coastal darkening which prevents the depth extension of key kelp species despite elongation of the open water period. This illustration is based on data from of Bartsch et al. (2016), Hop et al. (2012), and the present study.

Despite their ecological importance, Arctic kelp forests are largely understudied and the investigations in Kongsfjorden are a rare example of consistent quantitative monitoring that provide important data for predictions of the future Arctic (Bischof, Buschbaum et al., 2019; Filbee‐Dexter et al., 2019; Wernberg et al., 2019). It was therefore the objective of the current study to examine biomass and community composition in an Arctic kelp forest spanning over 25 years of Arctic warming. In 2021 we repeated the investigations from 1996/1998 (Hop et al., 2012) and 2012–2014 (Bartsch et al., 2016) at the Hansneset sampling site and complemented the existing time series (Figure 1). For our analysis of the historical datasets together with the newly collected data we posed several questions. (1) Did the upward shift of the local kelp forest documented between 1996/1998 and 2012/2013 continue in 2021? (2) How did the kelp forest structure, depth distribution, and demography develop over time at our study site? (3) Do mechanisms of biomass allocation to thallus parts vary between Arctic kelp species? We hypothesized that the overall community structure of the kelp forest has continuously changed according to local prevalent drivers. The observed alterations in macroalgal distribution and new investigations on structural differences between kelp species provide important insights for the key question, how kelp forests are affected in an Arctic fjord system that is influenced by warming, sea ice retreat and glacial melt.

## 2 MATERIALS AND METHODS

### 2.1 Study site

**FIGURE 2:**
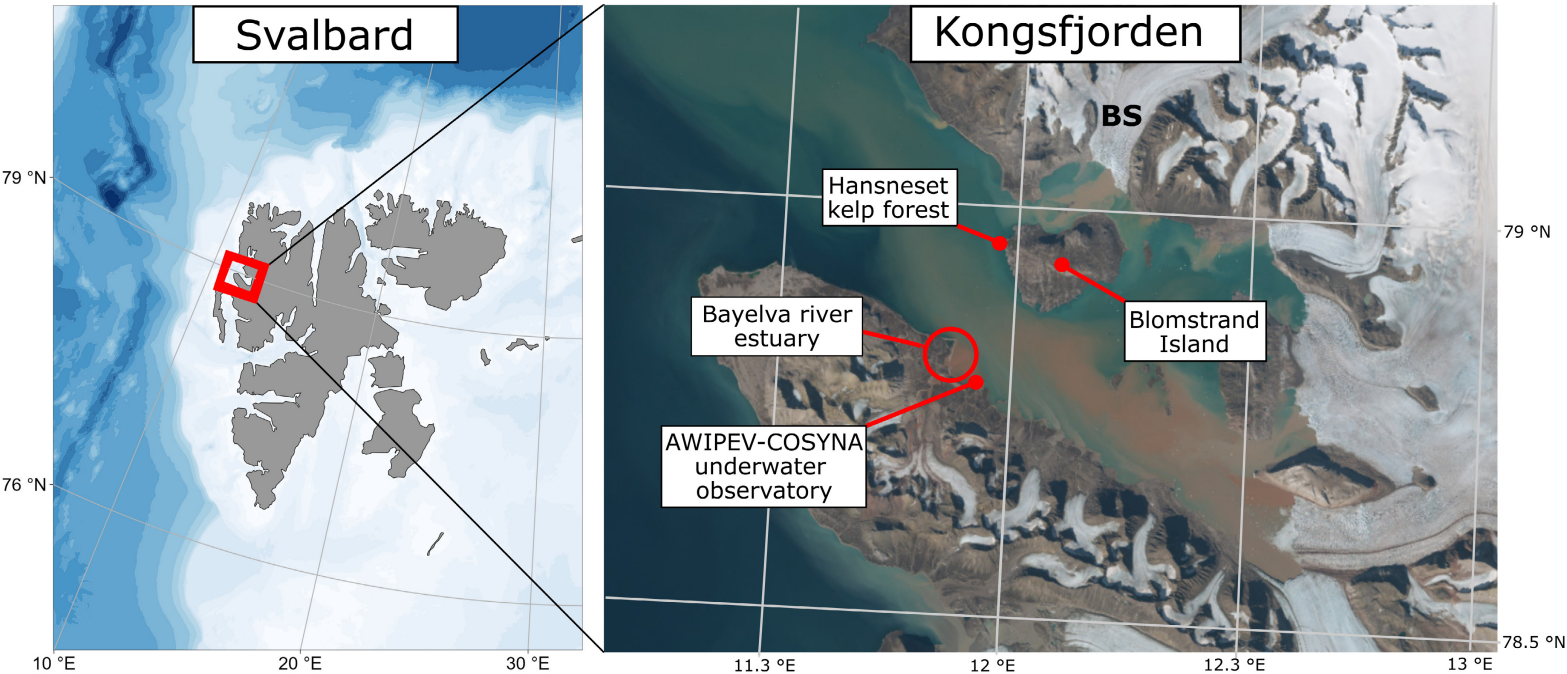
Study site of the depth transect at the Hansneset kelp forest and location of the AWIPEV‐COSYNA underwater observatory in Kongsfjorden, western Spitzbergen. BS, Blomstrandbreen; Svalbard Map, R package ggOceanMaps (Vihtakari, 2022) in R version 4.2.2 (“Innocent and Trusting,” R Core Team (2022). Kongsfjorden summer satellite image with sediment plumes: https://toposvalbard.npolar.no/; 08.01.2024, Norwegian Polar Institute.

Kongsfjorden is located on western Spitsbergen which is part of the Svalbard archipelago. Blomstrand is its largest island and situated in the center of the Arctic fjord. Until the early 1990s it was a peninsula and connected to the coast of Kongsfjorden by the retreating tidal glacier Blomstrandbreen (Burton et al., 2016; Svendsen et al., 2002). In summer 2021 the calving glacier front of Blomstrandbreen was right at the water edge and its base exclusively on land. Our study site Hansneset (78°59′17″N, 11°57.49″ E, for map see Figure 2) is located on the western side of Blomstrand island at about 5.9 km distance from this glacier but three more glaciers calve into the fjord. It is a moderately exposed hard‐bottom location (Voronkov et al., 2013) and consists of bedrock with scant sediment cover or pebbles down to 21 m. The sampling site slopes with a mostly steady angle of about 45°. The investigated depth transects followed the topography of the rock which is characterized by an escarpment of about 3 m height (increasing in steepness from north to south) right below the 10 m line. This reference point was marked by a permanent fastening bolt in the previous diving campaigns of 2012–2014 (Paar et al., 2016) and was used to ensure that the same area was sampled again in 2021. Since 2007, the site has remained ice free in winter and is relatively sheltered from occasional drift icebergs from the calving glaciers of the fjord (Lippert et al., 2001; Paar et al., 2016).

### 2.2 Study design

In the present study macroalgal biomass and species distribution investigations from 1996/1998 (Hop et al., 2012) and 2012–2014 (Bartsch et al., 2016) were repeated between 21 June and 15 July 2021 at the sampling site Hansneset (Table 1). To guarantee a comparable dataset and facilitate future monitoring at the study site, the survey methods followed the established protocols given in Bartsch et al. (2016) and Paar et al. (2016). In 2021, two successive diving campaigns were conducted of which the first was a visual census using semi‐quantitative categories on the depth distribution and abundance of dominant brown algae between 3 and 19 m depth. In contrast, the second campaign provided quantitative data through destructive sampling at 2.5, 5, 10, and 15 m depth and multiple associated measurements of the collected material (FW and dry weight (DW), leaf area index (LAI), and kelp demography). In both diving campaigns sampling depths were measured by a dive computer (Scubapro Digital 330) and afterwards corrected to chart datum (lowest astronomical tide) according to the local tide calendar (https://www.kartverket.no/en/at‐sea/).

**TABLE 1:** Overview of methods used in different campaigns in the Hansneset kelp forest and datasets included in the current time series analysis.

Campaign		1996/1998	2012–2014	2021
Reference	Category	Hop et al. (2012)	Bartsch et al. (2016)	This study
Depth distribution	Biomass dominant brown algae	Diving study and video transects 8–20 m depth in 1 m steps^a^ Two transects Combination of percentages cover classes and biomass data in semiquantitative cover classes	Diving study 8–20 m depth in 1 m steps Four transects Combination of relative frequency and presence/absence data in semiquantitative cover classes	Diving study 3–19 m depth in 1 m steps Five transects Combination of relative frequency and presence/absence data in semiquantitative cover classes
Biomass	All macroalgae	Fresh weight 0, 1.5 + 2.5, 5, 10, 15 m depth^a^ *n* = 2–4	Fresh and dry weight 0, 2.5, 5, 10, 15 m depth *n* = 3 or 6	Fresh and dry weight 0, 2.5, 5, 10, 15 m depth *n* = 3
Leaf area index	All macroalgae	_	2.5, 5, 10, 15 m depth *n* = 3	2.5, 5, 10, 15 m depth *n* = 3
Age structure and density	Kelp	_	2.5, 5, 10 m depth^a^ *n* = 3	2.5, 5, 10, (15) m depth *n* = 3
Blade, stipe, holdfast biomass allocation	Kelp	_	_	2.5, 5, (10) m depth *n* = 3

*Note*: In brackets: additional data collected in 2021 which could not be statistically evaluated due to absence of data in other years or most species.

^a^ Additional data were taken in 1996/1998 or 2012–2014 which have not been considered for comparative analysis between timepoints; for further details see Bartsch et al. (2016).

Macroalgal FW and the lower depth distribution of biomass dominant brown algae were analyzed across all three timepoints (this study, Bartsch et al., 2016; Hop et al., 2012). Furthermore, the LAI of biomass‐dominant species or groups was documented in 2021 and compared to 2012 data. The latter were sampled in 0.25 m^2^ frames and extrapolated to 1 m^2^ (Bartsch et al., 2016). Age structure and density of the kelp community was investigated along the Hansneset depth gradient (2.5–10 m) in summer 2013 and 2021 as a proxy for the stability of the environment. In 2021 a new aspect on kelp biomass allocation to thallus parts (holdfast, stipe and blade DW) was analyzed to reveal potential structural differences between the canopy‐forming species.

In the Arctic and sub‐Arctic, two morphologically very similar digitate kelp species (*Laminaria digitata* and *Hedophyllum nigripes*) may grow side by side and only DNA barcoding can reveal secure species identification (Dankworth et al., 2020), which has not been conducted here. Thus, this species complex is referred to as digitate kelps throughout this study, although the temperature requirements for growth and survival of both species are quite different (Franke et al., 2021).

### 2.3 Lower depth distribution of dominant brown algal species

The depth distribution of biomass dominant brown algae (Laminariales species, *Sacchoriza dermatodea*, and *Desmarestia* spp.) was visually investigated by scientific divers. In the multiple campaigns of the current time series, different divers were engaged who all underwent sampling protocol standardization training to recognize the species. In an adult stage all species are easy to differentiate by external morphology but even in juvenile form each species has unique features that are recognizable under water, for example, *S. dermatodea* has visible hairs in contrast to juvenile kelp. Young *Alaria* already has an obvious midrib, while *Saccharina* has a small elongate outline and a slightly thickened middle part in contrast to digitate kelps. In 2021, five parallel transects off the coastline were examined and coved the vertical gradient between 3 and 19 m depth (corrected to chart datum). All dives where video‐recorded and data were randomly quality checked afterwards by the authors. The overall sampling protocol followed Bartsch et al. (2016) and *Desmarestia* spp. includes *D. aculeata* and *D. viridis*. The target distance between transects was 5 m and transects were distributed over a horizontal width of approximately 30 m. A 1 × 1 m quadrat divided into four 50 × 50 cm subquadrats was placed on the ground or above the kelps at every depth meter along the transect and species occurrence was documented as attached frequency within each subquadrat. This resulted in a relative frequency of 0–4 per depth meter and transect. Additionally, the visual presence of the species in the close surroundings of each quadrat was documented. In summary, for each species this generated a presence score of 5 per replicate and an overall maximum score of 25 per depth level. In replicates with >50% coverage of kelps, it was not possible to place the quadrat on the ground. Here the depth was corrected by the mean height (78 cm) of the local kelp canopy (data not shown). In contrast, in 2014 only four transects were investigated with a maximum score of 20 per depth level and transect and Bartsch et al. (2016) established a scheme to compare their data to the publication of Hop et al. (2012). For the present study the score system of Bartsch et al. (2016) was adapted to a maximum score of 25 and the abundance of species was classified as rare (score 1–4), common (score 5–15), subdominant (score 16–20), and dominant (score 21–25). Due to the depth corrections to chart datum at 11 m only four transects were documented and in this case the original score system with a maximum score of 20 was used. In 1996/1998 these semi‐quantitative classes referred to a combination of biomass and %‐coverage values while in 2014 and 2021 a combination of relative frequency and presence/absence data was applied. Furthermore, investigated depth levels varied between time points (1996/1998: 8–20 m; 2014: 8–20 m; and 2021: 3–19 m).

### 2.4 Macroalgal biomass

For the quantitative diving campaign, a main transect was installed in the sublittoral off Hansneset, marking the vertical gradient between 2.5 and 15 m depth below chart datum. The same vertical gradient was secured by a bolt which had been drilled at 10 m in 2012 (Paar et al., 2016). Proceeding laterally ±15 m from this main transect, destructive sampling was performed by scientific diving at 2.5, 5, 10, and 15 m depth. In contrast to the previous studies (Bartsch et al., 2016; Paar et al., 2016) the horizontal transects were not permanently installed by drilling bolts in the rock bed but by fastening a diving reel to the fixed main transect. At each depth horizon the attached macroalgal biomass of all kelps and understory seaweeds was collected within randomly chosen 1 × 1 m quadrats (*n* = 3). Additional sampling was performed in the infralittoral fringe level (0 m) using 50 × 50 cm quadrats (*n* = 3) and data were extrapolated to 1 m^2^ before analysis. Algae were sampled in net bags and kept in seawater filled barrels for transport. In the laboratory, biomass samples were stored in flow through seawater tanks before further processing.

Algal material was sorted to species level, blotted dry with cotton towels and FW as well as DW was determined (Mettler Toledo PB3002_S/FACT Delta Range, Max 600 g/3100 g, *d* = 0.01 g/0.1 g, Germany). Biomass‐dominant species were organized in two categories as well as six groups (Table 2) and the historical datasets were reanalyzed accordingly for comparison. Some specimens of *Devaleraea ramentacea* were densely covered by brown *Elachista fucicola* epiphytes which could not be removed but were considered negligible relative to host biomass. Calcified coralline algae were present along the depth transect but were excluded from identification as in the previous studies.

**TABLE 2:** Organization of biomass dominant species in associated groups and categories which form the basis of the time series analysis presented in this manuscript.

Category	Group	Species
Kelps	*Alaria esculenta*	*Alaria esculenta*
	*Saccharina latissima*	*Saccharina latissima*
	Digitate kelps	*Laminaria digitata*
		*Hedophyllum nigripes*
Understory seaweeds	Other phaeophyceae	*Battersia arctica*
		*Chorda filum*
		*Chordaria flagelliformis*
		*Desmarestia aculeata*
		*Desmarestia viridis*
		*Dictyosiphon foeniculaceus*
		*Ectocarpus* sp.
		*Fucus distichus*
		*Halosiphon tomentosus*
		*Haplospora globosa*
		*Laminaria solidungula*
		Young *Laminaria* spp.
		*Pylaiella* sp.
		*Sacchorhiza dermatodea*
		*Scytosiphon* sp.
		*Chaetopteris plumosa*
	Rhodophyta	*Coccotylus truncatus*
		*Cystoclonium purpureum*
		*Devaleraea ramentacea*
		*Euthora cristata*
		*Odonthalia dentata*
		*Palmaria palmata*
		*Phycodrys rubens*
		*Plocamium cartilagineum*
		*Porphyra* sp.*/Pyropia* sp.
		*Ptilota* sp.
		*Rhodomela* sp.
		*Turnerella pennyi*
	Chlorophyta	*Acrosiphonia* spp.
		*Chaetomorpha melagonium*
		*Kornmannia leptoderma*
		*Spongomorpha* spp.
		*Ulva* sp.

Adult kelps were separated into holdfast, stipe and blade prior to weight measurements. Juvenile individuals with a stipe length ≤5 cm were weighed as whole individuals. DW of species was recorded after drying overnight at 70°C in an oven (Termaks, Series TS9000, Model TS9135). Due to logistical reasons, the DW could not be documented for every adult kelp specimen. For adult kelp DW representatives of each species and stipe length category were dried depending on their occurrence to represent the properties of the local kelps as realistic as possible [*Alaria esculenta*: *n* = 8 (5–15 cm, 31–50 cm, >80 cm); *n* = 10 (16–30 cm), *n* = 9 (51–80 cm); digitate kelps: *n* = 9 (5–15 cm), *n* = 10 (16–30 cm, 51–80 cm), *n* = 14 (31–50 cm), *n* = 3 (>80 cm); *Saccharina latissima*: *n* = 3 (5–15 cm), *n* = 8 (51–80 cm, >80 cm)]. The resulting regression formulas between FW and DW expressed high determination coefficients (majority *R* ^2^ > 0.9) and were used to calculate the DW of the remaining specimens (Appendix 1). All adult kelp individuals ≥2 years collected from 2.5 and 5 m depth were analyzed for holdfast, blade and stipe DW as well as blade:stipe DW ratio.

### 2.5 Leaf area index

The leaf area of all macroalgae collected from the quantitative sampling quadrats was determined from digital photographs (Canon EOS 600D) using black calibration quadrats and the image analysis software ImageJ (Schneider et al., 2012, Version 2.1.0). Single large specimens (e.g., kelps and kelp‐like brown algae) were recorded individually whereas all specimens of most understory algae per replicate were spread out and photographed together. According to Lüning (1969), the LAI is the leaf area normalized to 1 m^2^ ground. Samples from 0 m were not included in the LAI analysis. For very few adult kelp specimens (*A. esculenta* and digitate kelps: *n* = 4) the leaf area was not measured. In these rare cases, a regression formula between blade DW and leaf area of representatives of each species was calculated (Appendix 1) and applied to the respective specimens.

### 2.6 Age class distribution and density of kelps

To investigate the demographic structure of the Hansneset kelp forest, the age and density of kelp specimens was documented at 2.5, 5, and 10 m depth. The seasonal growth rhythm of kelps results in the formation of annual growth rings, which can be used for age determination in adult kelps [(Parke, 1948); for discussion see Bartsch et al. (2016)]. The minimum age of kelp individuals was determined by counting the annual growth rings of a thin cross section taken from the stipe just above the holdfast. Small delicate kelp individuals with ≤5 cm stipe length and specimens without growth rings were considered juveniles (<1 year old). For a minority of kelp individuals, the age was not recorded (*A. esculenta*: *n* = 4; digitate kelps: *n* = 21; *S. latissima*: *n* = 6) but calculated using a regression formula from stipe length and age following Rinde and Sjøtun (2005) (Appendix 1).

### 2.7 Turbidity and PAR

In situ data from the AWIPEV‐COSYNA underwater observatory in Ny‐Ålesund (78°55′48″N, 11°55′09″ E) were analyzed over time and used as a proxy for the general trend of light climate dynamics throughout Kongsfjorden. The underwater observatory is located on the opposite shore of Kongsfjorden, close to the estuary of the Bayelva River and about 6.8 km away from the macroalgal sampling site Hansneset (for map see Figure 2). Turbidity and PAR (photosynthetically active radiation) data were measured year‐round with a sampling frequency of 1 hz (1 s^−1^). For a detailed description of the AWIPEV‐COSYNA underwater observatory see Fischer et al. (2017, 2020). Briefly, the system comprises a land‐based FerryBox system equipped with various hydrographic sensors receiving water from a remote‐controlled underwater pump station at 11 m water depth. Additionally, a cable connected (fiber‐optic and 240 V power) underwater node system includes a fixed sensor carrier as well as a vertical profiling sensor elevator to the surface. For the analysis, data from a PAR (Sea‐Bird ECO‐PAR, installed in September 2016) and a turbidity sensor (Sea & Sun 90, installed in June 2012) were used, both positioned at 10 m (±1 m tide) water depth. Turbidity was measured in formazin turbidity unit (FTU) and PAR in μmol m^−2^ s^−1^. All sensors are maintained regularly in 1‐year interval. Data gaps due to system maintenance or a sensor failure were compensated by averaging all measurements within the 24‐h period to a single 24‐h mean value and subsequently averaging these daily means over 1 week. Weeks when no PAR and turbidity data were available at all were excluded and Appendix 2 shows the real number of available data weeks for all years. The focus of the present study is PAR and turbidity during the macroalgal growth season in the polar summer and therefore only data from week 8 in March to week 44 in October were included in the further analysis.

### 2.8 Statistical analyses

The statistical analyses were performed in R version 4.2.2 (“Innocent and Trusting,” R Core Team, 2022). The original data from 1996/1998 (https://doi.org/10.1594/PANGAEA.864321) and 2012–2014 (provided by I. Bartsch) were used for the time series analysis. Due to the logistical constraints of the intensive scuba diving campaign along the sublittoral gradient at Hansneset for all three time points of the time series, only a limited number of biomass quadrats were obtained per campaign [1996/1998: *n* = 2 (0 m); *n* = 3 (5–15 m); *n* = 4 (2.5 m); 2012/2013: *n* = 3 (0 and 15 m); *n* = 6 (2.5–10 m); 2021: *n* = 3]. The homogeneity of variances was tested using the Levene's test before each ANOVA. As the absence of a species or group at a certain depth level results in zero values for that depth, only relevant depth levels were included in the statistical tests.

#### 2.8.1 Time series analysis (1996/1998–2012/2013–2021)

Separate two‐factorial ANOVAs were performed to assess the effects of the fixed factors Time, Depth and their interaction for FW (log +1 transformed data) and LAI of the biomass‐dominant species or groups. When the ANOVA output revealed significant effects, a Tukey HSD post hoc test for uneven *n* was applied. In case homogeneity of variances could not be achieved by transformation but the results of the two‐factorial ANOVA were highly significant, a non‐parametric Kruskal–Wallis test followed by a pairwise Wilcoxon rank sum test was performed to test for differences between depth levels. This study focused on investigating changes over time and therefore the differences in species and group FW and LAI across depth levels in 2021 alone were not examined statistically.

#### 2.8.2 Age and density comparison 2013/2021

To investigate differences between timepoints and kelp species for the mean age and density per m^2^ at 2.5 and 5 m, separate two‐factorial ANOVAs were performed and significant effects were further investigated using a Tukey HSD post hoc test. For the analysis of mean density per m^2^, data were log +1 transformed to achieve homogeneity of variances. Juvenile specimens <1 year were excluded from the statistical analysis. Detailed data from 2013 were published as Table S3 in Bartsch et al. (2016).

#### 2.8.3 Adult kelp DW investigations in 2021

Individual holdfast, blade, and stipe DW, blade: stipe DW ratio was compared between kelp species and across the relevant depth levels (2.5–5 m) in 2021. Homogeneity of variances (Levene's test) and normal distribution (Shapiro–Wilk test) could not be achieved through data transformation. The effect of Species and Depth on the individual parameters were tested in separate non‐parametric Kruskal–Wallis tests and by pairwise Wilcoxon rank sum tests with Bonferroni correction to reveal differences between species.

#### 2.8.4 Turbidity and PAR time series

PAR and turbidity data were quality controlled according to an adapted ARGO standard for stationary sensors (Fischer et al., 2021; Waldmann et al., 2022). To analyze long‐term changes and trends over the sampling period, the residuals of the observed weekly PAR and turbidity values to the expected PAR and turbidity values were calculated, using the means of the observed weeks across all years as expected values. The trend‐analysis of PAR and turbidity over the sampling period was done by simple linear regression over time using the R base functions “lm” (R Core Team, 2019).

## 3 RESULTS

### 3.1 Seaweed biomass

#### 3.1.1 Seaweed biomass along the depth gradient in 2021

In 2021, a total of 21 biomass dominant macroalgal taxa (11 Phaeophyceae, 6 Rhodophyta, 4 Chlorophyta, >0.1 g FW m^−2^) were collected along the depth transect. In contrast to the previous studies, six macroalgal taxa were not biomass‐relevant anymore as they were either absent (Phaeophyceae: *Chorda filum*, *Haplospora globosa*, and *Laminaria solidungula*; Rhodophyta: *Cystoclonium purpureum* and *Odonthalia dentata*) or were only encountered in negligible amounts of <0.1 g FW (Phaeophyceae: *Battersia arctica*). Detailed FW and DW data of single species and sums of groups in 2021 are given in Appendices 3 and 4, respectively.

In the infralittoral zone (0 m) the macroalgal community was most diverse with 15 taxa present and understory seaweed FW, “Other Phaeophyceae” as well as Rhodophyta exhibited their maximum biomass. In contrast, the biomass peak of overall seaweed FW and kelp FW was located at 2.5 m. At 5 and 10 m the kelp species *A. esculenta* showed the highest biomass of all seaweeds. Because of the high *A. esculenta* FW, kelps were responsible for 95 and 56% of the overall seaweed FW at 5 and 10 m, respectively. At 15 m, the ground was almost exclusively covered with Rhodophyta and dominated by *Phycodrys rubens*, while *A. esculenta* was near to its lower distribution limit.

#### 3.1.2 Seaweed biomass timeseries (1996/1998–2012/2013–2021)

**FIGURE 3:**
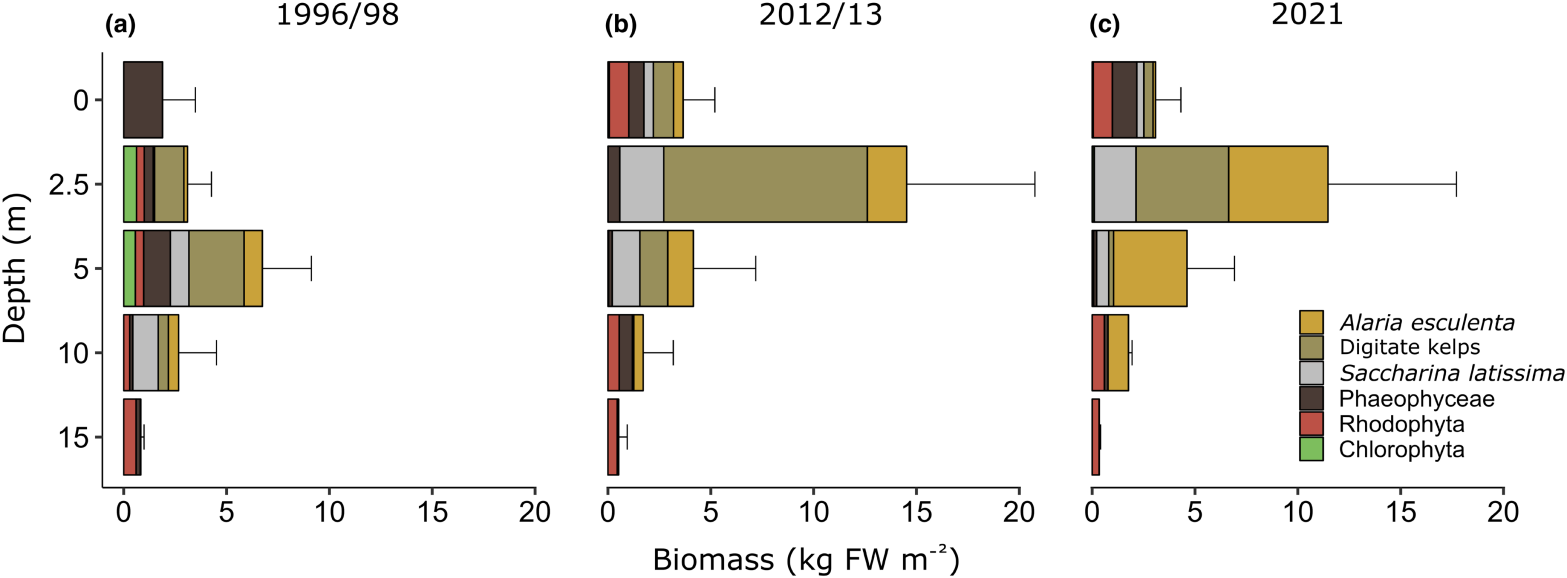
Fresh weight (FW) m^−2^ of biomass dominant macroalgal species and groups along the depth gradient at Hansneset, Kongsfjorden (Svalbard) over time (a, b, c) as indicated by different colors [mean ± SD; 1996/1998: *n* = 2 (0 m), *n* = 3 (5–15 m), *n* = 4 (2.5 m); 2012/2013: *n* = 3 (0 and 15 m), *n* = 6 (2.5–10 m); 2021: all depths *n* = 3; all *n*‐values refer to replicate quadrats].

Mean FW was compared over three time points (1996/1998, 2012/2013, and 2021) and across the biomass dominant species and groups at relevant depth levels. Figure 3 illustrates the change in FW over time and statistical results of separate two‐factorial ANOVAs for Time, Depth and Time × Depth interaction on FW are given in Table 3. Chlorophyta were only rarely recorded along the depth transect (1996/1998: 2.5 m, 5 m; 2012/2013: 0 m; 2021: 0 m, 2.5 m) and were therefore excluded from the statistical analysis.

**TABLE 3:** Results of two‐factorial ANOVA for Time, Depth, and their interaction on fresh weight of biomass‐dominant species or groups in the relevant depth levels.

Species/groups	Time		Depth		Time × depth	
All seaweeds (0–15 m)*	*F*_2,39_ = 1.457	ns	(*F*_4,39_ = 25.573)	**(*p* < .001)**	*F*_8,39_ = 3.303	***p* = .006**
All kelps (0–10 m)	*F*_2,33_ = 3.401	***p* = .045**	*F*_3,33_ = 19.883	***p* < .001**	*F*_6,33_ = 4.288	***p* = .003**
*Alaria esculenta* (2.5–10 m)	*F*_2,28_ = 4.585	***p* = .019**	*F*_2,28_ = 1.281	ns	*F*_4,28_ = 0.491	ns
Digitate kelps (2.5–5 m)	*F*_2,19_ = 4.408	***p* = .027**	*F*_1,19_ = 13.24	***p* = .002**	*F*_2,19_ = 2.879	ns
*Saccharina latissima* (0–10 m)	*F*_2,33_ = 0.509	ns	*F*_3,33_ = 3.118	***p* = .039**	*F*_6,33_ = 2.431	***p* = .047**
All understory seaweeds (0–15 m)	*F*_2,39_ = 0.853	ns	*F*_4,39_ = 4.434	***p* = .005**	*F*_8,39_ = 0.838	ns
‘Other Phaeophyceae’ (0–15 m)	*F*_2,39_ = 0.873	ns	*F*_4,39_ = 3.879	***p* = .01**	*F*_8,39_ = 0.747	ns
Rhodophyta (0–15 m)*	*F*_2,39_ = 0.361	ns	(*F*_4,39_ = 9.768)	**(*p* = <.001)**	*F*_8,39_ = 3.776	***p* = .002**

*Note*: 1996/1998: *n* = 2 (0 m); *n* = 3 (5–15 m); *n* = 4 (2.5 m); 2012/2013: *n* = 3 (0 and 15 m); *n* = 6 (2.5–10 m); 2021: all depths *n* = 3. In brackets: variances of residuals were still heterogeneous after transformation. Bold values indicate significant effects.

Abbreviation: ns, not significant.

* Significant depth effect confirmed by nonparametric Kruskal–Wallis test (*p* < .001).

Overall seaweed biomass along the depth transect (0–15 m) exhibited a significant Time × Depth interaction. When comparing 2021 data to earlier time points, the seaweed FW maximum at 2.5 m (11.5 kg FW m^−2^) was similar to 2012/2013 (14.5 kg FW m^−2^). In contrast, in 1996/1998 the seaweed FW maximum was approx. 50% lower (6.7 kg FW m^−2^) and at 5 m, explaining the significant Time × Depth interaction. The general pattern of seaweed FW distribution along the depth gradient was the same between 2012/2013 and 2021 but very different compared to 1996/1998. Additionally, seaweed FW was significantly affected by Depth [(2.5 = 5) > (5 = 0) > (0 = 10) > (10 = 15), *p* < .02, Tukey test].

As kelp FW (0–10 m) constituted most of the overall seaweed FW, it showed similar responses and exhibited a significant Time × Depth interaction. Between 1996/1998 and 2021 kelp FW at 2.5 m increased significantly 6.7‐fold from 1.7 to 11.4 kg FW m^−2^ (*p* = .03, Tukey test). Consequently, the increase and upward shift in kelp FW maximum from 5 to 2.5 m, which had already been observed between 1996/1998 and 2012/2013, remained the same in 2021. Furthermore, this relation is reflected in significant kelp FW differences with Depth [(2.5 m = 5 m) > (10 m = 0 m), *p* < .007, Tukey test]. In contrast to overall seaweed FW, kelp FW was also affected by Time [(2012/2013 = 2021) > (2021 = 1996/1998), *p* = .04, Tukey test].

Same as overall kelp FW, FW of the kelp species *Alaria esculenta* (2.5–10 m) changed significantly with Time and increased continuously [(2021 = 2012/2013) > (2012/2013 = 1996/1998), *p* = .017, Tukey test]. Similarly, FW of digitate kelps (2.5–5 m) was affected by Time, but Tukey Post hoc test did not reveal significant differences between years. Additionally, digitate kelp FW changed with Depth and was significantly higher at 2.5 m compared to 5 m (*p* = .002, Tukey test). Interestingly, FW of *Saccharina latissima* (0–10 m) showed a different performance than *A. esculenta* and digitate kelps as there was a significant Time × Depth interaction. In 1996/1998 *S. latissima* FW peaked at 10 m (1.2 kg FW m^−2^) in contrast to 2012/2013 and 2021 where the maximum was 1.6‐fold higher and recorded at 2.5 m (both years 2 kg FW m^−2^). Although *S. latissima* FW also changed significantly with Depth, these differences were not resolved via a Tukey Post hoc test.

Understory seaweeds FW (0–15 m) differed significantly between Depth levels [(0 m = 10 m) > (10 m = 15 m = 2.5 m = 5 m), *p* < .03, Tukey test]. Similarly, the group “Other Phaeophyceae” (0–15 m), which excludes adult kelps, was significantly affected by Depth [(0 m = 2.5 m) > (2.5 m = 10 m = 5 m = 15 m), *p* < .05, Tukey test]. In contrast, the FW of Rhodophyta (0–15 m) showed a significant Time × Depth interaction. While there were no biomass dominant Rhodophyta at 0 m in 1996/1998, the FW at this depth significantly increased to 0.95 and 0.93 kg FW m^−2^, respectively in 2012/2013 and 2021 (*p* = .04, Tukey test). Furthermore, the change in biomass distribution pattern over Time is reflected at 2.5 and 5 m where Rhodophyta FW was up to 93.5‐fold higher in 1996/1998 compared to 2012/2013 and 2021. Additionally, there was a significant effect of Depth [(0 m = 10 m = 15 m) > (5 m = 2.5 m), *p* < .03, Tukey test].

### 3.2 Leaf area index

#### 3.2.1 Seaweed LAI along the depth gradient in 2021

The LAI of biomass dominant species and groups in 2021 largely reflected their recorded FW and was different between species, groups and depth levels along the investigated transect (Appendix 5). All seaweed LAI reached its maximum at 2.5 m (LAI = 8.6), followed by 5 m as these depths were nearly exclusively inhabited by kelps (both depths 99%). In 2021 *A. esculenta* (LAI = 5) had the highest LAI of all seaweed species at 2.5 m, followed by digitate kelps and *S. latissima*. At 5 m *A. esculenta* LAI was still high with 3.2, while the LAI of digitate kelps and *S. latissima* severely declined below 0.3. The maximum LAI of understory seaweeds in 2021 was recorded at 10 m with a LAI of 1.6. At 15 m, Rhodophyta were the most prominent group contributing 99% to the all seaweed LAI.

#### 3.2.2 Seaweed LAI comparison between 2012 and 2021

**FIGURE 4:**
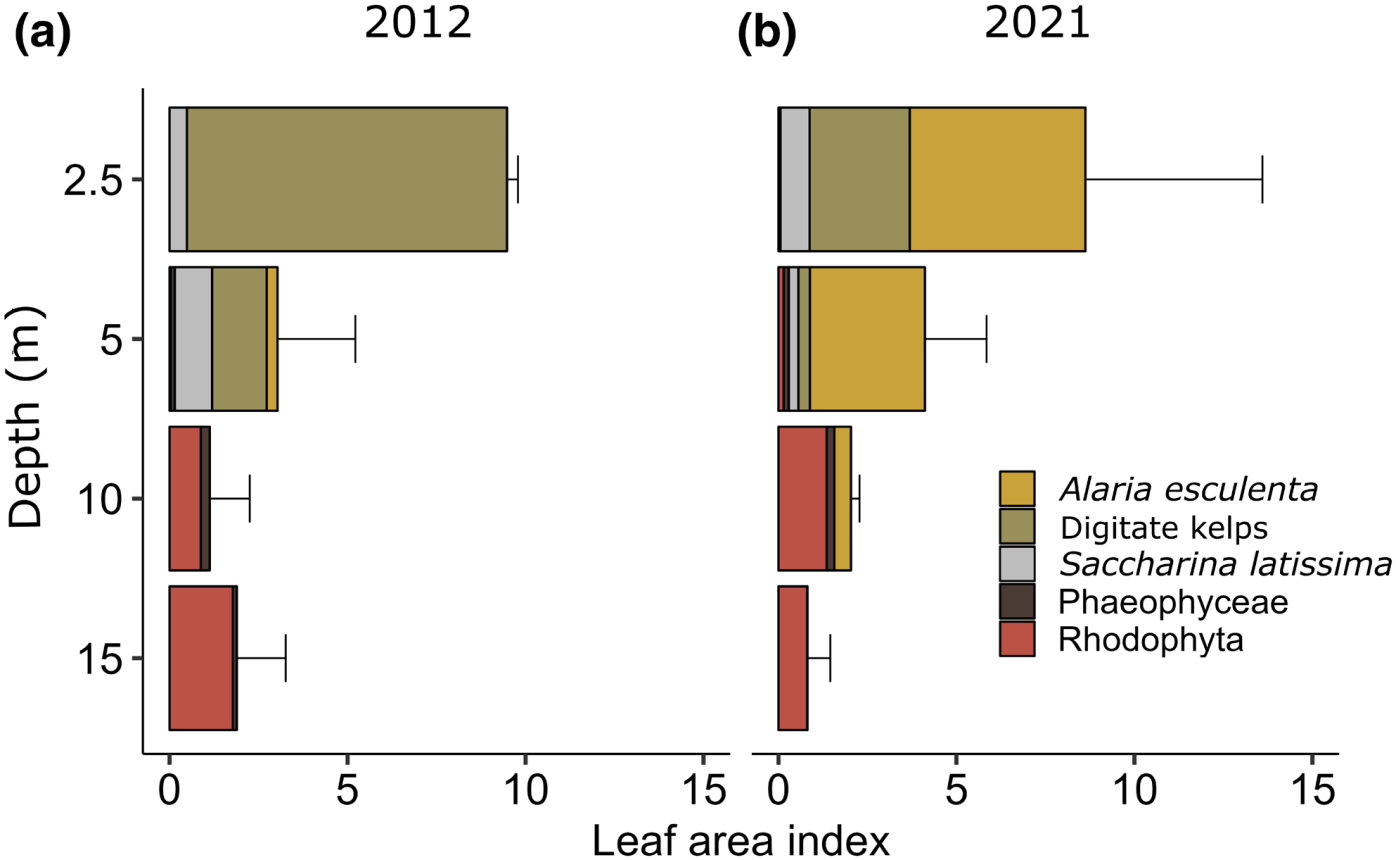
Leaf area index (LAI) of macroalgal species and groups along the depth gradient at Hansneset, Kongsfjorden (Svalbard) in summer 2012 (a) and 2021 (b) (mean ± SD, *n* = 3) as indicated by different colors. Original sample size differed between 2012 (0.25 m^2^) and 2021 (1 m^2^). In 2012 no kelps were documented in 0.25 m^2^ quadrats at 10 and 15 m though they were present at that depth.

The LAI was compared between 2012 and 2021 and across the species and groups at relevant depth levels. Detailed LAI data of species and groups in 2012 and 2021 are given in Appendix 5. Figure 4 illustrates the change in mean LAI between the 2 years and statistical results of the separate two‐factorial ANOVAs are given in Table 4. Overall there was no significant interactive Time × Depth effect in any of the investigated species or groups.

**TABLE 4:** Results of two‐factorial ANOVAs for Time, Depth, and their interaction on the leaf area index (LAI) of biomass‐dominant species and groups at the relevant depth levels (2012 and 2021: *n* = 3).

Species/groups	Time		Depth		Time × depth	
All seaweeds (2.5–15 m)	*F*_1,16_ = 0	ns	*F*_3,16_ = 17.005	***p* < .001**	*F*_3,16_ = 0.428	ns
All kelps (2.5–5 m)	*F*_1,8_ = 0	ns	*F*_1,8_ = 11.587	***p* = .009**	*F*_1,8_ = 0.308	ns
*Alaria esculenta* (5 m)^a^	*F*_1,4_ = 5.87	ns				
Digitate kelps (2.5–5 m)	*F*_1,8_ = 8.723	***p* = .018**	*F*_1,8_ = 15.809	***p* = .004**	*F*_1,8_ = 3.94	ns
*Saccharina latissima* (2.5–5 m)	*F*_1,8_ = 0.501	ns	*F*_1,8_ = 0	ns	*F*_1,8_ = 3.043	ns
All understory seaweeds (5–15 m)	*F*_1,12_ = 0.194	ns	*F*_2,12_ = 4.023	***p* = .046**	*F*_2,12_ = 1.528	ns
‘Other Phaeophyceae’ (5–15 m)	*F*_1,12_ = 0.208	ns	*F*_2,12_ = 1.505	ns	*F*_2,12_ = 0.233	ns
Rhodophyta (5–15 m)	*F*_1,12_ = 0.148	ns	*F*_2,12_ = 5.099	***p* = .025**	*F*_2,12_ = 1.716	ns

Abbreviation: ns, not significant.

*Note*: Bold values indicate significant effects.

^a^ In 2012 *Alaria esculenta* was only present at 5 m depth in the small frame size, therefore a one‐factorial ANOVA for the factor time was applied.

Digitate kelps (2.5–5 m) was the only group for which the LAI changed significantly over Time. Depth integrated LAI of digitate kelps was significantly higher in 2012 compared to 2021 (*p* = .018, Tukey test).

All other species and groups exhibited significant differences with Depth except for *Saccharina latissima* (2.5–5 m) and “Other Phaephyceae” (5–15 m). Time integrated total seaweed LAI (2.5–15 m) had its maximum at 2.5 m followed by a gradual decrease towards 15 m [2.5 m > (5 m = 10 m = 15 m), *p* < .002, Tukey test]. Similarly, time integrated LAI of all kelps (2.5–5 m) was significantly 2.6‐fold higher at 2.5 m compared to 5 m (*p* = .009, Tukey test). Time integrated digitate kelp LAI was significantly 84% lower at 5 m compared to 2.5 m (*p* < .004, Tukey test). Although time integrated understory seaweed LAI (5 – 15 m) changed significantly with Depth, the Tukey test did not reveal significant differences between depth levels. Time integrated Rhodophyta LAI (5–15 m) reached its maximum at 15 m which was significantly 14.7‐fold higher compared to 5 m [(15 m = 10 m) > (10 m = 5 m), *p* = .03, Tukey test].

### 3.3 Lower depth distribution of kelp and kelp‐like species over time

**FIGURE 5:**
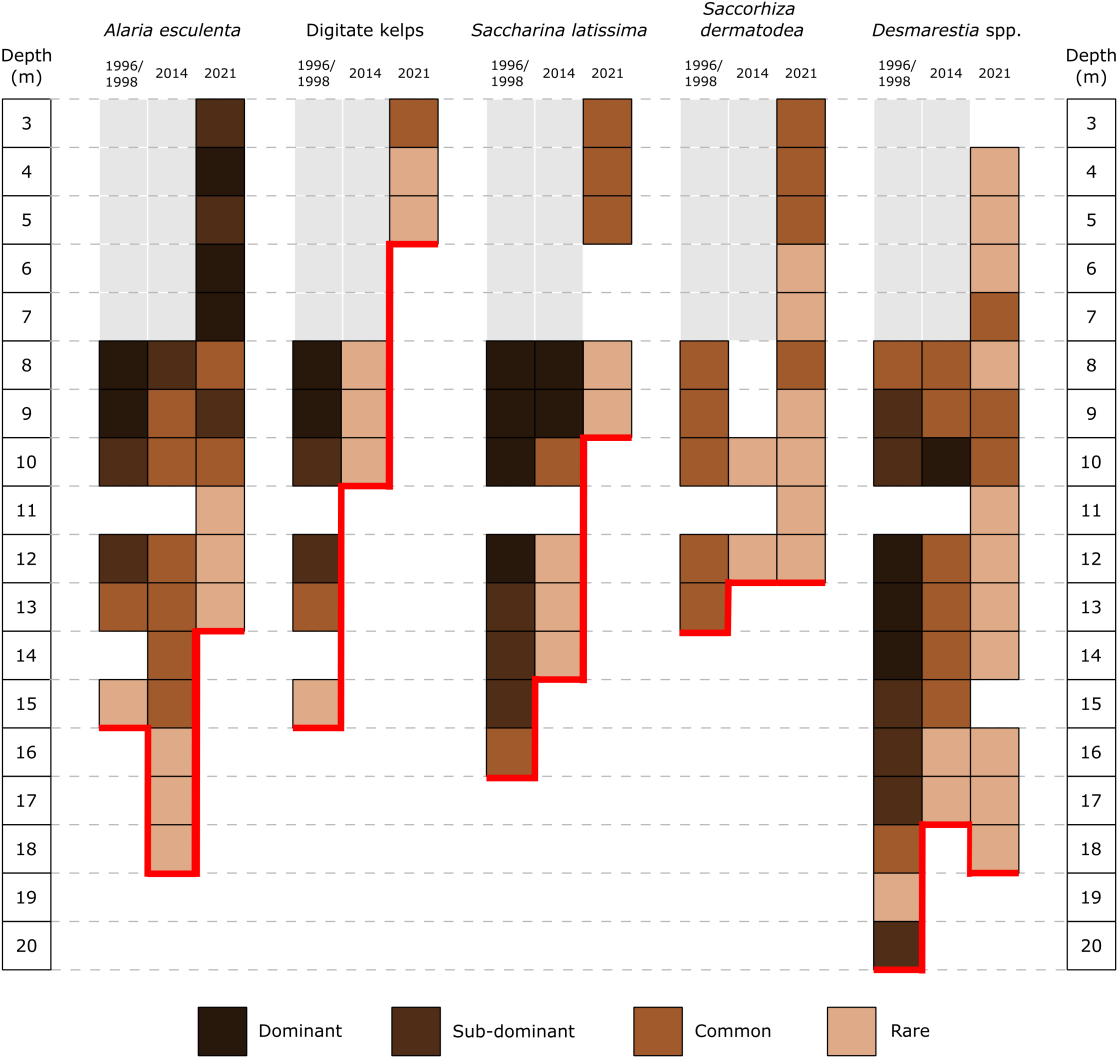
Changes of abundance and lower depth distribution (relative to chart datum) of biomass dominant brown algae species over time at Hansneset, Kongsfjorden (Svalbard). Absence of data between 2 and 7 m in 1996/1998 and 2014 is marked in gray. Changes in lower distribution limits are highlighted (red line) and semi‐quantitative abundance classes are indicated by color intensity.

An overall upward shift in lower depth distribution limit and a noticeable decrease in species abundance since 1996/1998 is evident for all investigated species (Figure 5). This change is especially prominent in digitate kelps and *S. latissima*. The lower distribution limit of digitate kelps decreased from 15 m in 1996/1998 to 10 m in 2014 to 5 m in 2021. Similarly, the recorded depth for lowest occurring *S. latissima* specimens decreased by 7 m over time from 16 m (1996/1998) over 14 m (2014) to 9 m (2021). For both taxa the shift in abundance which was already observed between 1996/1998 and 2014 continued in 2021 and these species were not classified as dominant or sub‐dominant any more.

In contrast to the other two kelp species, the lower depth distribution limit of *A. esculenta* remained relatively unchanged and varied between 15 m (1996/1998), 18 m (2014), and 13 m (2021). However, *A. esculenta* abundance decreased throughout the investigated time points as the species was sub‐dominant at 12 m in 1996/1998 while this abundance class moved upwards to 8 and 9 m in 2014 and 2021, respectively.

Similar to *A. esculenta*, the lowest distribution limits of *Saccorhiza dermatodea* and *Desmarestia* spp. only changed slightly over time. However, despite their stability in depth distribution, these species exhibited a decrease in abundance, which was especially strong in *Desmarestia* spp. While *Desmarestia* spp. had been mostly dominant to sub‐dominant in 1996/1998 between 20 and 9 m, these abundance classes were not recorded at any depth level in 2021.

### 3.4 Age structure and density of kelp species

#### 3.4.1 Relative age class distribution in 2021

**FIGURE 6:**
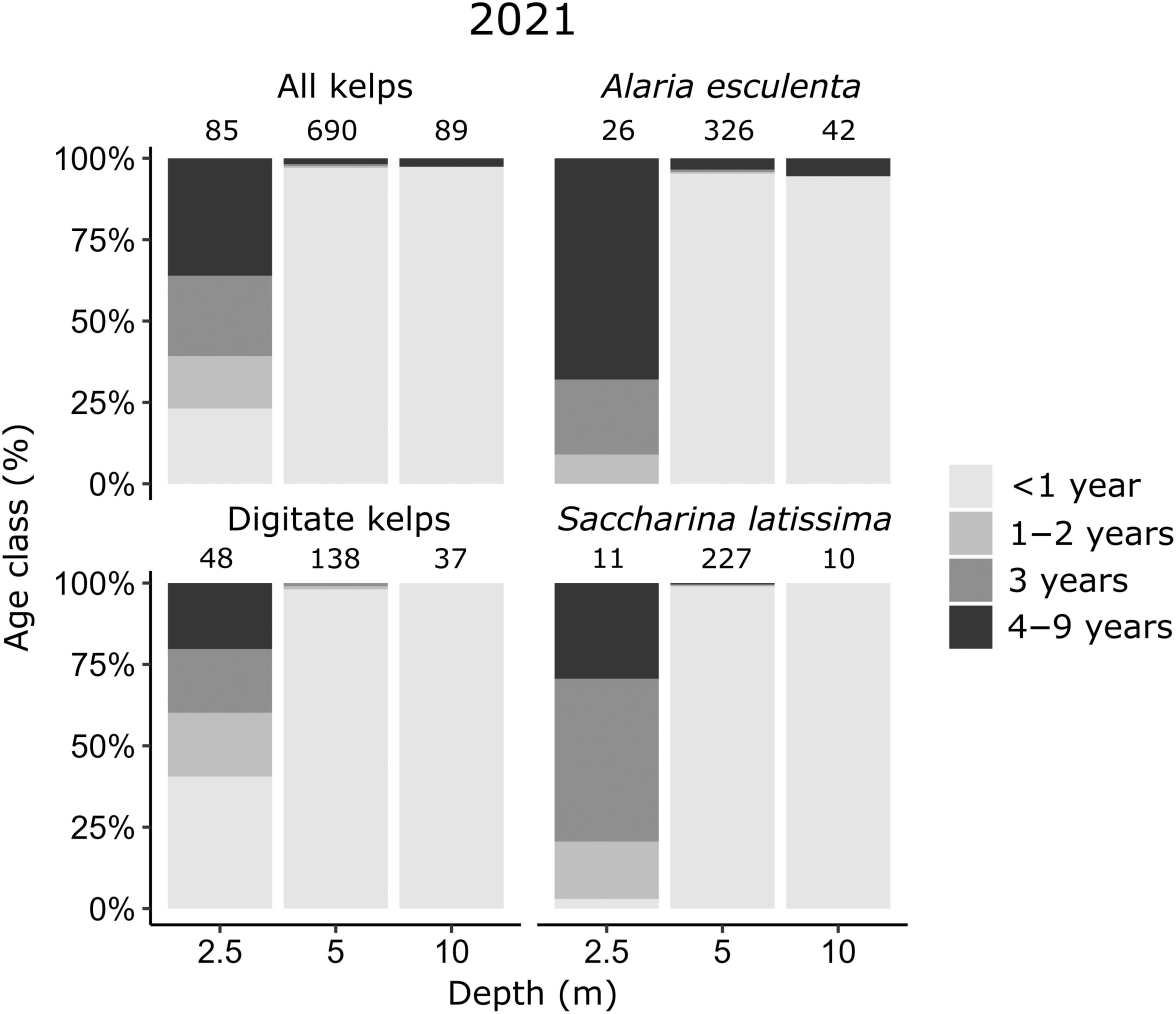
Relative age‐class distribution of all kelps and single kelp species along the depth gradient (2.5–10 m) at Hansneset, Kongsfjorden (Svalbard) in summer 2021. Numbers above stacked columns indicate the mean number of kelp individuals per m^2^ (*n* = 3). The age of kelp individuals was subsumed into four age classes indicated by color code.

The observed pattern of relative age class distribution and overall density of kelps changed considerably between the two time periods (Figure 6 and Appendix 6). In 2021, the relative abundance of adult kelps (≥1 year) peaked at 2.5 m in *A. esculenta*, digitate kelps, *S. latissima* and the overarching group “All kelps.” Furthermore, 2.5 m was the only depth in 2021 showing a balanced age structure with juveniles and all older age classes of kelps being present. At 5 m the relative abundance of juveniles sharply increased to ≥95% in all three kelp species and the overall kelp density reached its maximum with 690 ind. m^−2^. This pattern became even more pronounced at 10 m where 100% of all digitate kelps and *S. latissima* individuals were juveniles and *A. esculenta* was the only kelp species for which a few old individuals (5% 4–9 years) occurred. Interestingly, kelp densities at 2.5 m (primarily adults) and 10 m (primarily juveniles) were similar but at the same time noticeably lower compared to the density peak at 5 m (primarily juveniles).

#### 3.4.2 Age and density per m^2^ of adult kelps between 2013 and 2021

**FIGURE 7:**
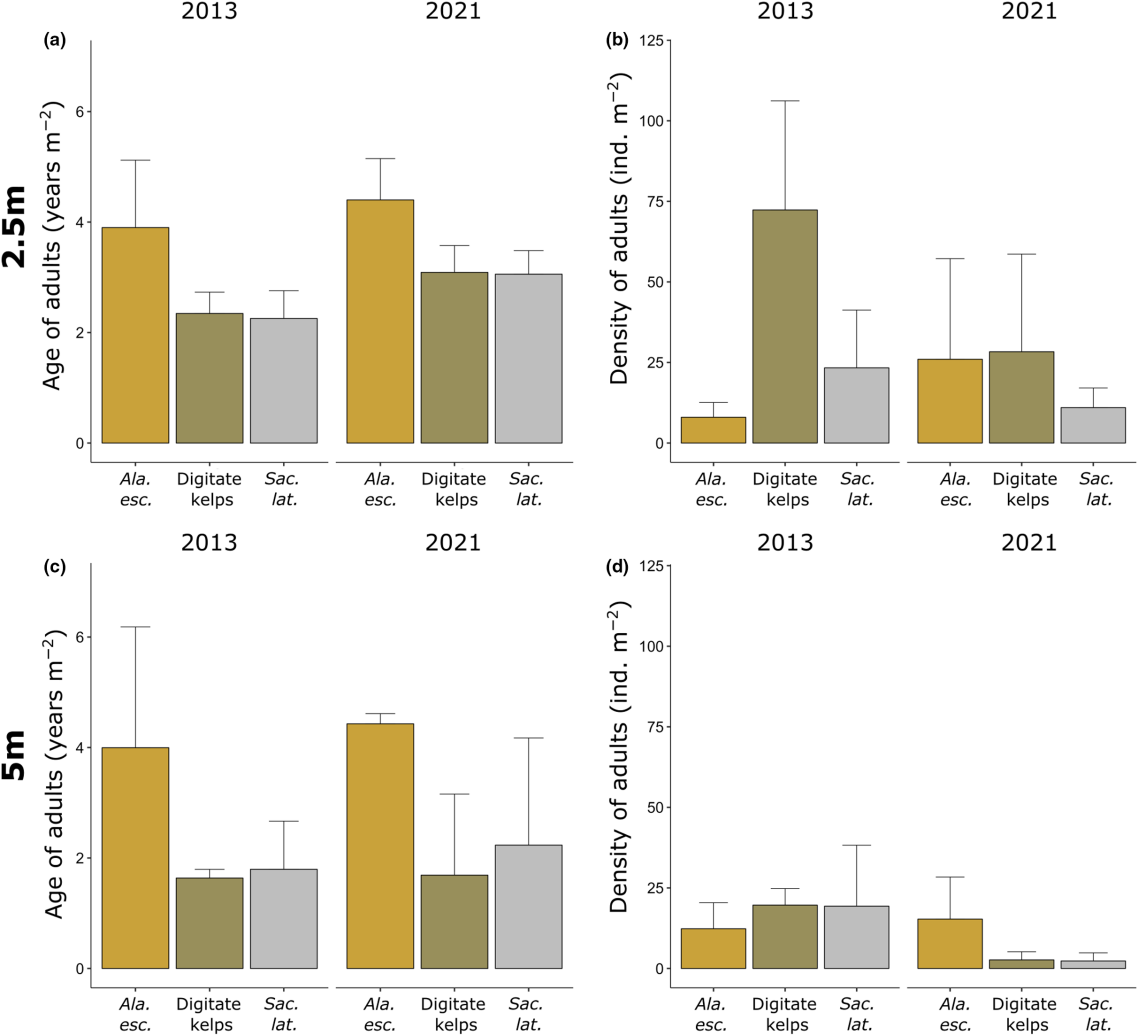
Age (a, c) and density (b, d) per m^2^ of adult kelps (≥ 1 year) at 2.5 m (a, b) and 5 m (c, d) at Hansneset, Kongsfjorden (Svalbard) between summer 2013 and 2021. Colors indicate kelp species (mean ± SD; 2013 and 2021: *n* = 3).

The age of adult kelps (≥1 year) at 2.5 and 5 m both was significantly affected by Species but not Time or Time × Species (Figure 7 and Table 5). At 2.5 m the time integrated age of *A. esculenta* (4.2 years m^−2^) was higher than of *S. latissima* and of digitate kelps (both 2.7 years m^−2^) [*A. esculenta* > (*S. latissima* = digitate kelps), *p* ≤ .01, Tukey test]. The species‐specific age differences became even more pronounced at 5 m as the time integrated age of *A. esculenta* remained at 4.2 years m^−2^, and was thereby more than double of *S. latissima* and digitate kelps [*A. esculenta* > (*S. latissima* = digitate kelps), *p* ≤ .05, Tukey test].

**TABLE 5:** Results of two‐factorial ANOVAs for Time, Species, and their interaction on the age and density per m^2^ of adult kelps (≥1 year) at 2.5 m and 5 m (2013 and 2021: *n* = 3).

Parameter	Depth	Time		Species		Time × species	
Age	2.5 m	*F*_1,12_ = 4.377	ns	*F*_2,12_ = 8.983	***p* = .004**	*F*_2,12_ = 0.080	ns
	5 m	*F*_1,12_ = 0.222	ns	*F*_2,12_ = 5.968	***p* = .02**	*F*_2,12_ = 0.038	ns
Density	2.5 m	*F*_1,12_ = 1.061	ns	*F*_2,12_ = 2.857	ns	*F*_2,12_ = 1.162	ns
	5 m	*F*_1,12_ = 10.411	***p* = .007**	*F*_2,12_ = 1.155	ns	*F*_2,12_ = 3.145	ns

Abbreviation: ns, not significant.

*Note*: Bold values indicate significant effects.

The density of adult kelps at 2.5 and 5 m did not change significantly with Time or Species and there was no interactive Time × Species effect (Figure 7 and Table 5). An interesting exception is the overall density of adult kelps at 5 m where species integrated density in 2013 (17.1 ind. m^−2^) was significantly higher compared to 2021 (6.8 ind. m^−2^).

### 3.5 Kelp biomass allocation in 2021

**FIGURE 8:**
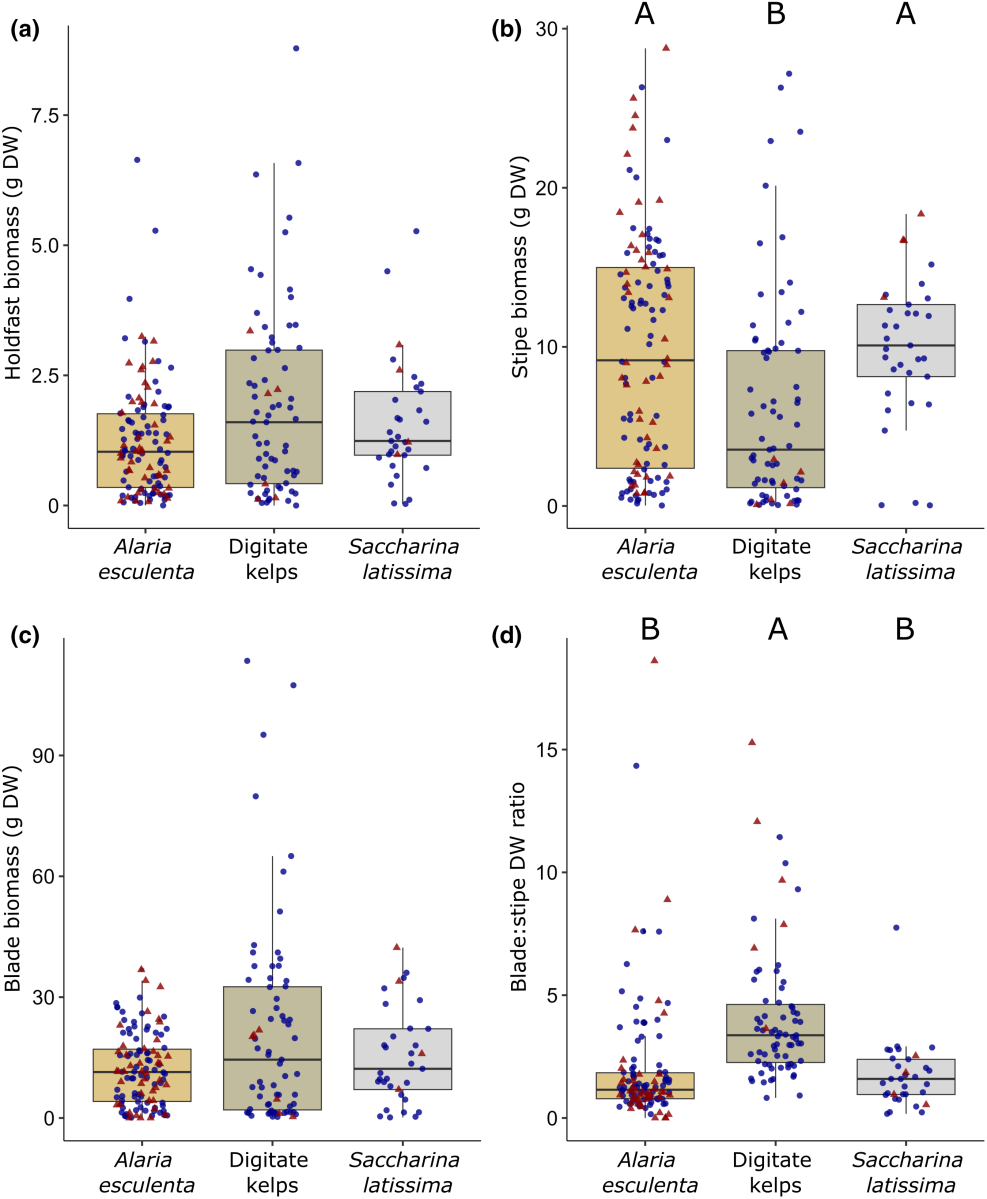
Individual holdfast (a), stipe (b), blade dry weight (DW) (c), and blade:stipe DW ratio (d) of adult kelps (≥ 2 years) collected from 2.5 and 5 m depth in Hansneset, Kongsfjorden (Svalbard) in summer 2021. Boxplots represent the median (50th percentile), the interquartile range (25th to 75th percentile), and whiskers the lower (5th) and upper (95th) percentile. Colors of boxplots indicate the three kelp species and single data points from 2.5 m (blue circles) and 5 m (red triangles) are presented. Significant differences between species are marked with different capital letters [non‐parametric Kruskal–Wallis test with pairwise Wilcoxon rank sum tests with Bonferroni correction, *A. esculenta: n* = 75 (2.5 m); *n* = 43 (5 m); digitate kelps: *n* = 65 (2.5 m); *n* = 6 (5 m); *S. latissima*: *n* = 29 (2.5 m); *n* = 4 (5 m)].

Holdfast, stipe and blade DW, and blade:stipe DW ratio of all adult kelps (≥2 years) collected in 2021 was compared between *A. esculenta*, digitate kelps and *S. latissima* from 2.5 and 5 m depth (Figure 8 and Table 6). Blade DW was not affected by Species or Depth. Interestingly, blade:stipe DW ratio of adult kelps was the only parameter that changed significantly with Species and Depth. Depth integrated blade:stipe DW ratio in digitate kelps was more than double compared to both *A. esculenta* and *S. latissima* (*p* < .001, Wilcoxon test). Species integrated blade:stipe DW ratio varied significantly between 2.5 and 5 m (*p* = .002, Wilcoxon test). In contrast to blade:stipe DW ratio, stipe DW and holdfast DW of adult kelp individuals were solely affected by Species but not Depth. Depth integrated stipe DW was significantly higher in *S. latissima* (9.9 ± 4.5 g DW) and *A. esculenta* (9.7 ± 7.2 g DW) compared to digitate kelps (6.2 ± 6.7 g DW; *p* < .002, Wilcoxon test). Although depth‐integrated holdfast DW varied significantly between Species, and was highest in digitate kelps (1.9 ± 1.8 g DW), the Wilcoxon test did not reveal the differences (all: mean ± SD).

**TABLE 6:** Results of non‐parametric Kruskal–Wallis test for Species and Depth on the dry weight (DW) of all adult kelp individuals (≥2 years) from 2.5 and 5 m depth collected in summer 2021 at Hansneset, Kongsfjorden (Svalbard).

Parameter	Depth			Species		
Holdfast DW	*X* ^2^ = 0.365	dF = 1	ns	*X* ^2^ = 6.142	dF = 2	***p* < .05**
Stipe DW	*X* ^2^ = 2.566	dF = 1	ns	*X* ^2^ = 16.608	dF = 2	***p* < .001**
Blade DW	*X* ^2^ = 0.179	dF = 1	ns	*X* ^2^ = 2.969	dF = 2	ns
Blade: stipe DW ratio	*X* ^2^ = 9.170	dF = 1	***p* = .002**	*X* ^2^ = 67.011	dF = 2	***p* < .001**

*Note*: *A. esculenta: n* = 75 (2.5 m); *n* = 43 (5 m); digitate kelps: *n* = 65 (2.5 m); *n* = 6 (5 m); *S. latissima*: *n* = 29 (2.5 m); *n* = 4 (5 m). Bold values indicate significant effects.

Abbreviation: ns, not significant.

### 3.6 Turbidity and PAR

**FIGURE 9:**
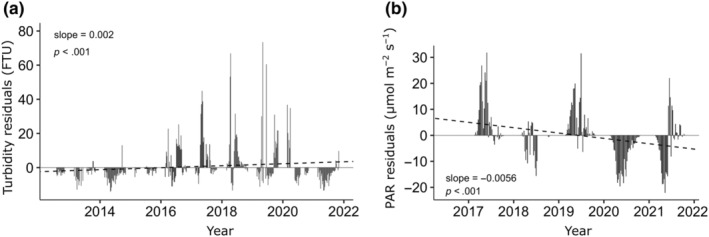
Long‐term change in turbidity (a) and PAR (b) at the AWIPEV‐COSYNA underwater observatory off Ny‐Ålesund, Kongsfjorden. Weekly means originate from the daily vertical water column profiles recorded between 11 and 1 m depth (±tide). The deviation of the observed weekly PAR and turbidity values to the expected PAR and turbidity values is shown. The expected value (zero line) was calculated as the mean of the calendar week across all years. A simple linear regression model (dashed line) was used to analyze the trend in PAR and turbidity change over the years. Only the photosynthetic active period (summer light condition) from week 8 (March) to week 44 (October) was considered.

Turbidity significantly increased over time with an average numerical increase of 0.104 FTU units per year (Figure 9a and Appendix 7, slope per week = 0.002 FTU × average numbers of weeks per year = 52). Even more prominent as the absolute average increase in turbidity per year, however, was the change in the extreme values of turbidity. Starting in 2016, the positive residuals in turbidity significantly increased until 2020 with maximal values in 2019 of up to 70 FTU. In 2021, lower values, similar to the pre‐2016 phase were observed. Contrary to the turbidity, the average PAR values per week significantly decreased over time from 2017 to 2021 with a numerical value of −0.29 μmol m^−2^ s^−1^ per year (Figure 9b and Appendix 7). Similar to turbidity, not only the absolute numerical PAR values per week changed but also the seasonal character of the phases changed to lower photon fluence rates. This became especially prominent in 2020 when PAR values lower than the expected mean were measured over the entire year.

## 4 DISCUSSION

Our study indicates that Arctic kelp species differently responded to the strong environmental changes which have occurred in Kongsfjorden (e.g., Bischof, Buschbaum et al., 2019; Maturilli et al., 2019; Pavlova et al., 2019; Payne & Roesler, 2019; Tverberg et al., 2019). Since the last investigation 2012–2014, the kelp forest structure and demography along the depth transect noticeably changed in 2021. Digitate kelps and *Saccharina latissima* retreated to the uppermost depth level (2.5 m) and *Alaria esculenta* became the dominant seaweed species down to 10 m water depth. This shows that within the relatively short time period of 7 years, a community which used to be dominated by digitate kelps transformed into an *Alaria esculenta* kelp forest. Our time series also documented that all investigated kelp and kelp‐like species, including *A. esculenta*, decreased their depth distribution and abundance along the depth gradient across the years. Additionally, the depth of the biomass maximum decreased and the whole kelp forest shallowed considerably. The uppermost depth level of 2.5 m remained the only depth in 2021 at which all three prevailing kelp species still had a balanced age structure with juveniles and adults of different age classes characterizing a mature kelp forest while at greater depths juvenile kelps became predominant. The observed changes in depth extension and alterations in species dominance over time are associated with a shift in structure and function as we show that each kelp species exhibits a species‐specific mechanism in biomass allocation.

One major abiotic factor that changed over the investigated time period as a consequence of global warming is summer seawater temperature (Payne & Roesler, 2019). In July and August 1997 temperatures varied around 4°C at Hansneset in the water column down to 20 m (Hanelt et al., 2001). In contrast, between 2016 and 2021 the monthly median ocean temperature at 11 m depth was 6.1°C in August at the AWIPEV‐COSYNA underwater observatory (Gattuso et al., 2023). Except for Arctic endemic *Laminaria solidungula* which is particularly impacted by warming waters (Roleda, 2016; Tom Dieck, 1992), experimental laboratory studies have shown that most kelp species present in Kongsfjorden are capable of coping well with increasing water temperatures if they do not surpass 10°C (Diehl & Bischof, 2021; Franke et al., 2021; Tom Dieck, 1993). *Laminaria solidungula* is a rare species for Kongsfjorden, which had only been present with very few and small individuals at Hansneset in the past (Bartsch et al., 2016; Hop et al., 2012), and was not encountered anymore in our quantitative 2021 samples. However, in other Svalbard fjords (Ronowicz et al., 2020; Wiktor Jr et al., 2022) and Arctic sites (Kreissig et al., 2021; Muth et al., 2021) *L. solidungula* is still present and forms wider stands.

Rising temperatures modify the physiological responses and demands of organisms which ultimately can affect interspecific competition in Arctic kelp forests (Doney et al., 2012; Molis et al., 2019). But temperature may only partially explain the observed shift in species dominance, especially as interactions between species and with other environmental factors seem to be very complex and only partially understood. The group “digitate kelps” comprises warm‐temperate *Laminaria digitata* and cold‐temperate to Arctic *Hedophyllum nigripes*. Both *A. esculenta* and *H. nigripes* have a preference to cold temperatures in gametogenesis and sporophyte growth and survival (Franke et al., 2021; Kraan, 2020; Munda & Lüning, 1977; Zacher et al., 2019) whereas *L. digitata* and *S. latissima* have a wider temperature range and up to 5°C higher temperature optima (Diehl et al., 2024; Franke et al., 2021; Bolton & Lüning, 1982; Tom Dieck, 1992). While a laboratory study with juvenile sporophytes indicated a competitive advantage of *A. esculenta* over *L. digitata* when both species were co‐cultivated at 5°C and 9°C due to faster growth rates in *A. esculenta* (Zacher et al., 2019), this relationship is unknown for *H. nigripes*. As *H. nigripes* was much more abundant than *L. digitata* at 5 and 7.5 m in 2015 at Hansneset (Dankworth et al., 2020), the current decrease of digitate kelps from deeper sites is possibly due to a retreat of *H. nigripes*. In eastern Canada field studies showed that warmer winters markedly reduced the abundance of *H. nigripes* over *L. digitata* (Longtin & Saunders, 2016), indicating that *H. nigripes* is especially sensitive to warming. However, in Kongsfjorden winter temperatures still stay below 1°C (Gattuso et al., 2023). Even though *S. latissima* is especially able to acclimate to rising temperature conditions (Diehl & Bischof, 2021), this did not seem to be relevant for shaping the prevailing kelp forest structure at our study site.

The reduction of ice scouring may explain the documented shallowing of the multi‐annual kelp forest and biomass increase at our study site over time. Ice scouring is a major disruptive force in near‐shore Arctic habitats and can largely prevent the colonization of shallow hard substrates by macroalgae (Barnes & Conlan, 2007; Campana et al., 2009; Molis et al., 2019). The reduction of physical disturbance with warming is considered a major diver of kelp forest expansion in the future Arctic (Assis et al., 2022). Even though weaker than in 2012–2014, also in 2021 the observed variation in macroalgal biomass possibly was a consequence of intermediate ice scouring. Especially at 2.5 m the samples were largely heterogeneous, as one replicate was dominated by *A. esculenta*, one by digitate kelps while the third was a mix of both and *S. latissima* but contained fewer adult kelps, overall indicating differential physical disturbance. Macroalgal species generally differ in their acclimation potential and capacity to recover from physical disturbance, which is considered an underlying mechanism shaping Arctic community composition (Fricke et al., 2008). Species succession and recolonization in areas cleared by disturbance appear to be much slower in polar regions compared to temperate latitudes (Barnes & Conlan, 2007; Bonsell & Dunton, 2021; Beuchel & Gulliksen, 2008; Dunton et al., 1982). As shallow Arctic sites (2.5 m) are prone to constant disturbances and seasonal fluctuations in abiotic stressors they may permanently stay in intermediate successional stages that allow species coexistence (Beuchel & Gulliksen, 2008; Campana et al., 2009; Molis et al., 2019). Over time possibly a climax community will develop in the undisturbed deeper areas of the depth transect (5, 10, and 15 m) that is constant in biomass and composition of biomass relevant species but information on long‐term algal succession in polar benthic habitats is scarce (Beuchel & Gulliksen, 2008; Campana et al., 2009). In colder Arctic fjords ice scouring is presumably more intense and thus macroalgal biomass maximizes at depths between 5 and 15 m. This was reported at our study site in 1996/1998 (Hop et al., 2012), on the colder east coast of Svalbard (Wiktor Jr et al., 2022) or in the Eastern Canadian Arctic (Filbee‐Dexter et al., 2022).

Interestingly, despite the biomass stability at 5 and 10 m the density of adult kelps decreased substantially here since 2013 (Bartsch et al., 2016) while juvenile kelps became dominant. Recruitment and colonization rates, and thus overall community development, in Arctic kelp forests are strongly controlled by local abiotic factors like underwater light availability (Bonsell & Dunton, 2021; Campana et al., 2009). Our results indicate that irradiance conditions to support multi‐annual kelp stands may have deteriorated in recent years possibly only allowing germination and gametogenesis of kelp gametophytes but not further growth of sporophytes. Gametogenesis and sporophyte formation may take place at very low irradiance conditions (e.g., Laeseke et al., 2019; Lüning & Dring, 1975; Tom Dieck, 1992) while kelp growth is only saturated at one order of magnitude higher irradiances (e.g., Fortes & Lüning, 1980). We therefore assume that the potentially positive effect of reduced shading by a decrease in sea ice cover for kelp depth expansion (Castro de la Guardia et al., 2023; Filbee‐Dexter et al., 2022; Krause‐Jensen et al., 2012) might get devalued at sites with diminishing water transparency. The latter is a consequence of increasing glacial melt and coastal erosion (Bonsell & Dunton, 2018; Castro de la Guardia et al., 2023; Niedzwiedz & Bischof, 2023; Payne & Roesler, 2019) as has been observed in Kongsfjorden. Increased light attenuation and the competition for light (Dean et al., 1989; Reed & Foster, 1984) was therefore likely the major structuring mechanism shaping kelp demography. This fits the circumstance that Kongsfjorden experiences the phenomenon of “coastal darkening” as water transparency considerably decreased between 1997 and 2019 (Konik et al., 2021). Glaciers on Svalbard (including Kongsfjorden area), retreated substantially over time as a response to warming summer temperatures (Geyman et al., 2022). The increasing subglacial meltwater discharge of the five sea‐terminating glaciers is suspected to be the main source of the increasing sedimentation in Kongsfjorden (Svendsen et al., 2002). We confirmed this darkening trend with in situ measurements from the AWIPEV‐COSYNA underwater observatory and provide evidence that the turbidity of the water column has increased over time while light availability for macroalgal photosynthesis declined. The observed lower turbidity values in 2021 may have occurred due to the comparatively colder spring and summer temperatures in the marine Kongsfjorden ecosystem (https://dashboard.awi.de/?dashboard=2847). However, the location of our sensors at the outflow of the Bayelva River is 6 km distant to our macroalgal study site and can therefore only serve as a proxy for the general trend of decreasing light levels with rising glacial melt throughout Kongsfjorden (Figure 2).

In addition to the changed kelp demography indicating reduced availability of light, we observed a considerable decrease in species dominance and depth expansion of major brown algae. Thus, we hypothesize that the deterioration of the underwater light conditions is one major driver for the observed structural shifts in the kelp forest community and is likely also due to increased sediment resuspension into the fjord as similarly observed in Alaska (Bonsell & Dunton, 2018). Overall, the observed changes may be a result of the physiological effects of a darkening water column in combination with the physical stress of sediment particles covering photosynthetic active surfaces (Chapman et al., 2002; Niedzwiedz & Bischof, 2023). Ecophysiological studies showed that an increase in turbidity and sedimentation can have negative effects on photosynthetic rates of adult kelps (Roleda et al., 2008), germination capacity of spores as well as recruitment success of juvenile kelps (Roleda, 2016; Zacher et al., 2016) and thus on the presence and overall resilience of Arctic kelps (Tatsumi & Wright, 2016). Interactions between kelp species appear to have changed along the turbidity stress gradient from competitive in benign conditions at 2.5 m to less competitive in the harsh environments of the deeper areas following the Stress Gradient Hypothesis (Bennett et al., 2015; Bertness & Callaway, 1994). Supporting our in situ data, *A. esculenta* spore germination and sporophyte recruitment were reported to be less susceptible to sediment loading than those of *L. digitata* and *S. latissima* (Zacher et al., 2016). Especially under the current abiotic conditions in Kongsfjorden, with low underwater light availability and temperatures mostly <7°C, *A. esculenta* is better adapted and has a competitive advantage over *S. latissima* (Niedzwiedz & Bischof, 2023). These kelp species specific differences in tolerance to the occurred increase in turbidity and sedimentation over time in Kongsfjorden is reflected in the noticeable upward shift of *S. latissima* and digitate kelps in contrast to *A. esculenta* that still exhibited high numbers of juveniles and a few adult specimens at 5 and 10 m.

Kelps are basal habitat‐formers that enhance positive species interactions, for example, facilitation cascades with secondary foundation species (Gribben et al., 2019). Kelp forests serve as biodiversity hot spots (Elliott Smith & Fox, 2022), therefore structural changes may have profound implications for all associated species and coastal ecosystem functioning. We showed that Arctic kelp species possess different strategies in biomass allocation to perennial structures of holdfast, stipe, and annual formation of blades. Adult digitate kelps invested most biomass in their holdfast and blades, *S. latissima* and *A. esculenta* into their stipe. When abundance and dominance relationships in a kelp forest change over time, its 3D structure and therefore the habitat conditions for epifaunal communities shift accordingly (Lippert et al., 2001; Niklass, 2022; Paar et al., 2016). Epifaunal biodiversity is highest on kelp holdfasts compared to blades and lowest on stipes (Włodarska‐Kowalczuk et al., 2009). Consequently, the decrease in digitate kelps and their large holdfasts indicates the loss of an important habitat structure that is not substituted by *A. esculenta*. Also higher trophic levels like fish are influenced by kelp bottom coverage (Brand & Fischer, 2016) and climate‐driven shifts in kelp abundance might substantially impact local food webs (Smale, Teagle et al., 2022).

Kelp forest dynamics in Arctic fjord ecosystems are affected by the complex alterations in the numerous abiotic factors related to climate change (Schlegel et al., 2023) and it is consequently difficult to pinpoint a certain stressor. Similar to our interpretation, Fragkopoulou et al. (2022) suggest that light is one of the main drivers shaping subtidal macroalgal communities. Furthermore, the reduction in underwater light availability and an associated decline in vertical distribution of macroalgae were reported from many other sites worldwide including temperate (Smith et al., 2022) and polar locations (Bonsell & Dunton, 2018; Deregibus et al., 2016; Ronowicz et al., 2020). Regardless of the warming with associated reduction in sea‐ice and darkening of Kongsfjorden, the seaweed community at Hansneset persisted over time, although with a depth reduced kelp zone. This indicates a general resilience of macroalgae against the local stressors and altered physiochemical conditions. The three sampling points of this time series are only snapshots but they continuously documented adult kelps and all major brown algal species, even in 2021. Overall rocky shores along Arctic coasts and fjord systems are dynamic systems and different sites are individually exposed to environmental factors. Thus, this local study cannot be extrapolated to the whole Arctic but may provide clues how cryosphere loss may alter seaweed communities. More long‐term studies of kelp forest structure development at different Arctic sites are needed to improve our understanding on how interactive effects of abiotic and biotic drivers impact species composition and biomass distribution in polar macroalgal communities (Campana et al., 2009; Krumhansl et al., 2016; Molis et al., 2019).

## 5 CONCLUSION

In this novel Arctic time series, we report considerable changes in kelp forest community structure and demography over the past 25 years in Kongsfjorden on Svalbard which are likely the result of global climate change. In contrast to proposed assumptions (Castro de la Guardia et al., 2023; Krause‐Jensen et al., 2012) we observed a decrease of depth expansion of the investigated kelp forest despite its release from ice scouring and shading by thick sea ice coverage. The documented shallowing of the kelp forest reflects a decline of key foundation species exposed to low light conditions with potentially large impacts on all associated higher trophic levels. Simultaneously, the observed kelp forest biomass increase over time highlights the promotion of macroalgal growth predicted to occur with Arctic warming (Assis et al., 2022; Krause‐Jensen & Duarte, 2014). As kelp communities represent complex marine ecosystems our investigation serves as one of the rare case studies at polar sites that quantifies macroalgal forest parameters in relation to the consequences of cryosphere loss, to facilitate future predictions for wider stretches of Arctic coastline. We propose that in Arctic fjord systems influenced by strong sediment runoff, kelp forest depth extension will decline further as long as glaciers retreat and coastal darkening intensifies. At the same time, kelp forests at shallow depths might flourish on the condition that temperatures and solar radiation are not exceeding tolerance limits. Overall, the rapidly shrinking cryosphere affects marine primary producers and their highly valuable ecosystem services with unknown consequences for a changing future Arctic.

## AUTHOR CONTRIBUTIONS

**Luisa Düsedau:** Conceptualization (supporting); data curation (lead); formal analysis (lead); investigation (equal); validation (lead); visualization (lead); writing – original draft (lead); writing – review and editing (lead). **Stein Fredriksen:** Investigation (supporting); supervision (supporting); validation (supporting); writing – review and editing (supporting). **Markus Brand:** Investigation (supporting); resources (equal); writing – review and editing (supporting). **Philipp Fischer:** Data curation (supporting); formal analysis (supporting); investigation (supporting); resources (equal); visualization (supporting). **Ulf Karsten:** Writing – review and editing (supporting). **Kai Bischof:** Funding acquisition (lead); supervision (supporting); writing – review and editing (supporting). **Amanda Savoie:** Supervision (supporting); writing – review and editing (supporting). **Inka Bartsch:** Conceptualization (lead); investigation (equal); project administration (lead); supervision (lead); validation (supporting); writing – original draft (supporting); writing – review and editing (supporting).

## CONFLICT OF INTEREST STATEMENT

There is no conflict of interest, all permits regarding sampling were given by Norwegian authorities (Sysselmannen Svalbard).

## Data Availability

The data supporting the conclusions of this article are available on PANGAEA: Düsedau, Luisa; Fredriksen, Stein; Brand, Markus; Fischer, Philipp; Karsten, Ulf; Bischof, Kai; Savoie, Amanda; Bartsch, Inka: Quantitative macroalgae survey and kelp morphometry data collected at Hansneset, Kongsfjorden, Spitsbergen in 2021. PANGAEA, https://doi.pangaea.de/10.1594/PANGAEA.963565. Düsedau, Luisa; Fredriksen, Stein; Brand, Markus; Fischer, Philipp; Karsten, Ulf; Bischof, Kai; Savoie, Amanda; Bartsch, Inka: Semi‐quantitative macroalgae survey on abundance and lower depth distribution of dominant brown algal species collected at Hansneset, Kongsfjorden, Spitsbergen in 2021. PANGAEA, https://doi.pangaea.de/10.1594/PANGAEA.963583. Düsedau, Luisa; Fredriksen, Stein; Brand, Markus; Fischer, Philipp; Karsten, Ulf; Bischof, Kai; Savoie, Amanda; Bartsch, Inka: Kelp demography and density along the depth gradient monitored at Hansneset, Kongsfjorden, Spitsbergen in 2021. PANGAEA, https://doi.pangaea.de/10.1594/PANGAEA.963439. Düsedau, Luisa; Fredriksen, Stein; Brand, Markus; Fischer, Philipp; Karsten, Ulf; Bischof, Kai; Savoie, Amanda; Bartsch, Inka: Kelp demography and density along the depth gradient monitored at Hansneset, Kongsfjorden, Spitsbergen in 2013 and 2021. PANGAEA, https://doi.pangaea.de/10.1594/PANGAEA.964589. Düsedau, Luisa; Fredriksen, Stein; Brand, Markus; Fischer, Philipp; Karsten, Ulf; Bischof, Kai; Savoie, Amanda; Bartsch, Inka: Mean fresh weight of macroalgae collected at Hansneset, Kongsfjorden, Spitsbergen in 2012, 2013 and 2021. PANGAEA, https://doi.pangaea.de/10.1594/PANGAEA.964587. Düsedau, Luisa; Fredriksen, Stein; Brand, Markus; Fischer, Philipp; Karsten, Ulf; Bischof, Kai; Savoie, Amanda; Bartsch, Inka: Mean leaf area of macroalgae collected at Hansneset, Kongsfjorden, Spitsbergen in 2012 and 2021. PANGAEA, https://doi.pangaea.de/10.1594/PANGAEA.964590. PAR and turbidity data from the AWIPEV‐COSYNA Underwater Observatory in Ny‐Ålesund supporting the findings of this study are available on the AWI dashboard. https://dashboard.awi.de/?dashboard=3865.

## APPENDIX 1

### 1.1

Regression formulas for the calculation of the following relationships per kelp species (whole thallus) or per parts of them (blade/stipe/holdfast) for fresh weight (FW): dry weight (DW), stipe length (SL): age, DW: leaf area index (LAI), whole thallus fresh weight (WT FW): DW.

**Table:**

Species	*x*	*y*	Formula	*R* ^2^	*n*
*Digitate kelps*					
Holdfast	FW	DW	*y* = 0.2021*x* – 0.035	.9856	46
Stipe	FW	DW	*y* = 0.1177*x* – 0.0146	.9954	46
	SL	Age	*y* = 1.0728*x* ^0.3231^	.8552	116
Blade	FW	DW	*y* = 0.1803*x* – 2.6118	.9689	46
	DW	LAI	*y* = 46.991*x* + 211.6	.9278	46
Whole thallus	FW	DW	*y* = 0.164*x* – 2.7325	.9842	46
*Alaria esculenta*					
Holdfast	FW	DW	*y* = 0.1521*x* + 0.0382	.9745	41
Stipe	FW	DW	*y* = 0.1137*x* + 0.4676	.9747	43
	WT FW	DW	*y* = 0.0421*x* – 0.3275	.8056	42
	SL	Age	*y* = 0.9277*x* ^0.3877^	.7037	142
Sporophyll	FW	DW	*y* = 0.2126*x* + 0.5854	.8925	29
Blade	FW	DW	*y* = 0.1452*x* – 0.2493	.9802	42
	WT FW	DW	*y* = 0.0652*x* + 0.9214	.8871	41
	DW	LAI	*y* = 120.79*x* + 313.89	.8893	42
Whole thallus	FW	DW	*y* = 0.1497*x* + 0.0431	.9672	42
*Saccharina latissima*					
Holdfast	FW	DW	*y* = 0.2429*x* – 0.316	.9373	18
Stipe	FW	DW	*y* = 0.0899*x* + 0.952	.9156	18
	WT FW	DW	*y* = 0.0365*x* + 1.9363	.8235	18
	SL	Age	*y* = 0.8107*x* ^0.3197^	.7725	42
Blade	FW	DW	*y* = 0.1452*x* – 0.2746	.9556	18
	WT FW	DW	*y* = 0.0847*x* – 2.7372	.8538	18
Whole thallus	FW	DW	*y* = 0.1255*x* + 0.0329	.9448	18

## APPENDIX 2

### 2.1

Number of weeks with PAR and turbidity data available in each year obtained from the AWIPEV‐COSYNA (Coastal Observing Systems of the Northern and Arctic Seas) underwater observatory off Ny Ålesund, Kongsfjorden. Only the summer period from week 8 (March) to week 44 (October) was considered.

**Table:**

	PAR	Turbidity
Year	*n*	*n*
2012		14
2013		24
2014		37
2015		20
2016	3	37
2017	37	31
2018	24	37
2019	37	34
2020	36	22
2021	37	37

## APPENDIX 3

### 3.1

Mean rounded fresh weight (g m^−2^) ± standard deviation of all seaweed species and summarized values of species groups at Hansneset, Kongsfjorden in summer 2021 (*n* = 3) at 0, 2.5, 5, 10, and 15 m depth. Data from 0 m depth referred to 0.25 m^−2^ frames but have been normalized to 1 m^2^ here.

**Table:**

Species	0 m	2.5 m	5 m	10 m	15 m
All kelps	911 ± 505	11,363 ± 6194	4394 ± 2392	994 ± 123	0.9 ± 0.9
*Alaria esculenta*	122 ± 71	4828 ± 6192	3571 ± 2318	994 ± 123	0.9 ± 0.9
Digitate kelps	449 ± 234	4504 ± 6688	238 ± 222	0	0
*Saccharina latissima*	341 ± 587	2031 ± 1562	586 ± 508	0	0
‘Other Phaeophyceae’	1191 ± 542	10 ± 18	149 ± 171	179 ± 248	3 ± 3
*Chordaria flagelliformis* and *Dictosiphon* sp.	164 ± 192	0	0	0	0
*Desmarestia aculeata*	0	0	92 ± 101	157 ± 249	2 ± 3
*Desmarestia viridis*	0	0	0	0.8 ± 1.4	0.6 ± 1
Ectocarpales	476 ± 351	0	0	0	0
*Fucus distichus*	549 ± 101	0	0	0	0
*Halosiphon tomentosus*	0	0.9 ± 1.6	37 ± 59	0.2 ± 0.4	0
*Saccorhiza dermatodea*	0	10 ± 17	16 ± 14	0	0
*Scytosiphon* sp.	1 ± 0.9	0	0	0	0
Young *Laminaria* spp.	0	0	4 ± 3	21 ± 6	0
Rhodophyta	927 ± 462	4 ± 4	66 ± 59	589 ± 79	335 ± 69
*Devaleraea ramentacea*	810 ± 430	0	0	0	0
*Euthora cristata*	0	0	3 ± 3	4 ± 1	6 ± 0.1
*Palmaria palmata*	40 ± 40	0	0	0	0
*Phycodrys rubens*	0	0.2 ± 0.2	14 ± 11	446 ± 113	317 ± 51
*Ptilota* spp.	2 ± 3	4 ± 4	49 ± 47	140 ± 37	18 ± 24
*Rhodomela* sp.	76 ± 98	0	0	0	0
Chlorophyta	52 ± 18	86 ± 149	0	0	0
*Acrosiphonia* sp. and *Spongomorpha* sp.	23 ± 22	86 ± 149	0	0	0
*Chaetomorpha melagonium*	0.6 ± 0.6	0	0	0	0
*Kornmannia leptoderma*	24 ± 34	0	0	0	0
*Ulva* sp.	4 ± 7	0	0	0	0
All understory seaweeds	2169 ± 1021	100 ± 171	215 ± 178	769 ± 273	338 ± 72
All seaweeds	3080 ± 1233	11,463 ± 6247	4609 ± 2308	1763 ± 177	339 ± 71

## APPENDIX 4

### 4.1

Mean rounded dry weight (g m^−2^) ± standard deviation of all seaweed species and summarized values of species groups at Hansneset, Kongsfjorden in summer 2021 (*n* = 3) at 0, 2.5, 5, 10, and 15 m depth. Data from 0 m depth referred to 0.25 m^−2^ frames but have been normalized to 1 m^2^ here.

**Table:**

Species	0 m	2.5 m	5 m	10 m	15 m
All kelps	123 ± 58	1632 ± 849	634 ± 323	170 ± 18	0.2 ± 0.2
*Alaria esculenta*	19 ± 12	703 ± 871	531 ± 318	170 ± 18	0.2 ± 0.2
Digitate kelps	62 ± 29	674 ± 1021	32 ± 31	0	0
*Saccharina latissima*	42 ± 73	254 ± 184	71 ± 61	0	0
‘Other Phaeophyceae’	189 ± 164	0.6 ± 1.1	17 ± 17	31 ± 47	0.4 ± 0.5
*Chordaria flagelliformis* and *Dictosiphon* sp.	30 ± 40	0	0	0	0
*Desmarestia aculeata*	0	0	12 ± 11	29 ± 47	0.4 ± 0.5
*Desmarestia viridis*	0	0	0	0.1 ± 0.2	0.1 ± 0.1
Ectocarpales	77 ± 76	0	0	0	0
*Fucus distichus*	82 ± 73	0	0	0	0
*Halosiphon tomentosus*	0	0.1 ± 0.1	3.2 ± 5	0.02 ± 0.03	0
*Saccorhiza dermatodea*	0	0.6 ± 1	1 ± 0.9	0	0
*Scytosiphon* sp.	0.05 ± 0.05	0	0	0	0
Young *Laminaria* spp.	0	0	0.3 ± 0.3	2.3 ± 0.4	0
Rhodophyta	131 ± 128	0.9 ± 1	13 ± 11	115 ± 20	66 ± 14
*Devaleraea ramentacea*	113 ± 126	0	0	0	0
*Euthora cristata*	0	0	0.5 ± 0.5	0.5 ± 0.1	0.01 ± 0.01
*Palmaria palmata*	5.2 ± 7.9	0	0	0	0
*Phycodrys rubens*	0	0.1 ± 0.1	2.6 ± 2	90 ± 27	62 ± 10
*Ptilota* spp.	0.1 ± 0.1	0.8 ± 0.9	9.6 ± 9	25 ± 7	3.8 ± 4.9
*Rhodomela* sp.	12.8 ± 17.4	0	0	0	0
Chlorophyta	9.3 ± 3.5	0.1 ± 0.2	0	0	0
*Acrosiphonia* sp. and *Spongomorpha* sp.	4.4 ± 4.1	0.1 ± 0.2	0	0	0
*Chaetomorpha melagonium*	0.2 ± 0.2	0	0	0	0
*Kornmannia leptoderma*	4.1 ± 6.6	0	0	0	0
*Ulva* sp.	0.6 ± 1.1	0	0	0	0
All understory seaweeds	330 ± 289	1.6 ± 2.2	30 ± 19	146 ± 48	66 ± 15
All seaweeds	453 ± 309	1633 ± 849	664 ± 309	316 ± 34	66 ± 15

## APPENDIX 5

### 5.1

Mean rounded leaf area index ± standard deviation of all seaweed species and summarizing species groups at Hansneset, Kongsfjorden in summer 2012 and 2021 (*n* = 3) at 2.5, 5, 10, and 15 m depth. Data from 2012 refer to 0.25 m^2^ frames but have been normalized to 1 m^2^ here.

**Table:**

Species	Year	2.5 m	5 m	10 m	15 m
All kelps	2012 2021	9.5 ± 0.3 8.6 ± 5	3 ± 2 4 ± 2	0 0.5 ± 0.2	0 0.005 ± 0.004
*Alaria esculenta*	2012 2021	0 5 ± 6	0.3 ± 0.4 3.2 ± 2	0 0.5 ± 0.2	0 0.005 ± 0.004
Digitate kelps	2012 2021	9 ± 0.6 2.8 ± 4	1.5 ± 1.4 0.3 ± 0.3	0 0	0 0
*Saccharina latissima*	2012 2021	0.5 ± 0.7 0.8 ± 0.7	1 ± 0.4 0.27 ± 0.2	0 0	0 0
‘Other Phaeophyceae’	2012 2021	0 0.04 ± 0.07	0.11 ± 0.08 0.15 ± 0.15	0.25 ± 0.25 0.22 ± 0.29	0.1 ± 0.1 0.006 ± 0.006
*Desmarestia aculeata*	2021	0	0.02 ± 0.02	0.14 ± 0.22	0.005 ± 0.006
*Desmarestia viridis*	2021	0	0	0.004 ± 0.007	0.002 ± 0.003
*Halosiphon tomentosus*	2021	0.005 ± 0.008	0.08 ± 0.13	0.0006 ± 0.001	0
*Saccorhiza dermatodea*	2021	0.035 ± 0.06	0.01 ± 0.024	0	0
Young *Laminaria* spp.	2021	0	0.03 ± 0.02	0.07 ± 0.08	0
Rhodophyta	2012 2021	0 0.02 ± 0.01	0.03 ± 0.06 0.14 ± 0.14	0.88 ± 0.87 1.35 ± 0.24	1.8 ± 1.3 0.8 ± 0.6
*Euthora cristata*	2021	0	0.006 ± 0.004	0.03 ± 0.04	0.0002 ± 0.0004
*Phycodrys rubens*	2021	0.0005 ± 0.0004	0.06 ± 0.06	0.99 ± 0.26	0.75 ± 0.6
*Ptilota* spp.	2021	0.015 ± 0.013	0.077 ± 0.078	0.33 ± 0.08	0.06 ± 0.07
All understory seaweeds	2012 2021	0 0.055 ± 0.08	0.15 ± 0.08 0.29 ± 0.2	1.1 ± 1.1 1.57 ± 0.47	1.9 ± 1.4 0.8 ± 0.6
All seaweeds	2012 2021	9.5 ± 0.3 8.6 ± 5	3 ± 2 4 ± 2	1 ± 1 2 ± 0.2	2 ± 1 0.8 ± 0.6

## APPENDIX 6

### 6.1

Mean rounded density ± standard deviation per age class of kelp species per m^2^ at Hansneset, Kongsfjorden in summer 2021 (*n* = 3) at 2.5, 5, 10, and 15 m depth.

**Table:**

Species	2.5 m	5 m	10 m	15 m
*Alaria esculenta*				
Juveniles	1 ± 2	311 ± 383	40 ± 21	3 ± 5
1	0	0	0	0
2	2 ± 3	2 ± 2	0	0
3	6 ± 7	2 ± 2	0	0
4	4 ± 7	5 ± 4	1 ± 1	0
5	7 ± 9	4 ± 5	<1 ± 1	0
6	5 ± 8	2 ± 1	<1 ± 1	0
7	1 ± 1	1 ± 1	0	0
8	0	0	1 ± 1	0
9	<1 ± 1	0	0	0
*Digitate kelps*				
Juveniles	19 ± 13	135 ± 140	37 ± 10	0
1	2 ± 3	0	0	0
2	8 ± 8	1 ± 2	0	0
3	9 ± 6	1 ± 1	0	0
4	7 ± 13	0	0	0
5	2 ± 3	0	0	0
6	<1 ± 1	0	0	0
*Saccharina latissima*				
Juveniles	<1 ± 1	224 ± 344	10 ± 9	0
1	<1 ± 1	0	0	0
2	2 ± 1	1 ± 1	0	0
3	6 ± 5	1 ± 1	0	0
4	3 ± 3	1 ± 1	0	0
5	<1 ± 1	<1 ± 1	0	0

## APPENDIX 7

### 7.1

Absolute weekly turbidity and PAR values recorded at the AWIPEV‐COSYNA (Coastal Observing Systems of the Northern and Arctic Seas) underwater observatory off Ny‐Ålesund, Kongsfjorden. Data for the winter period from week 45 (November) to week 7 (February) not shown
